# Functional 2D Nanoplatforms Alleviate Eosinophilic Chronic Rhinosinusitis by Modulating Eosinophil Extracellular Trap Formation

**DOI:** 10.1002/advs.202307800

**Published:** 2024-03-13

**Authors:** Zhaoxu Tu, Ming Liu, Changyi Xu, Yi Wei, Tong Lu, Yongqiang Xiao, Hongxia Li, Shuaiyin Zhang, Xinran Xie, Jian Li, Weiping Wen

**Affiliations:** ^1^ Department of Otolaryngology The Sixth Affiliated Hospital Sun Yat‐sen University Guangzhou Guangdong 510655 China; ^2^ Department of Otolaryngology The First Affiliated Hospital Sun Yat‐sen University Guangzhou Guangdong 510655 China; ^3^ Biomedical Innovation Center The Sixth Affiliated Hospital Sun Yat‐sen University Guangzhou Guangdong 510655 China; ^4^ Department of Clinical Laboratory The Sixth Affiliated Hospital Sun Yat‐sen University Guangzhou Guangdong 510655 China; ^5^ ENT institute Eye & ENT Hospital Fudan University Shanghai 201114 China

**Keywords:** cell‐free DNA, eosinophil extracellular traps, eosinophilic chronic rhinosinusitis, functional 2D nanoplatforms, linear molecular architecture

## Abstract

The therapeutic outcomes of patients with eosinophilic chronic rhinosinusitis (ECRS) remain unsatisfactory, largely because the underlying mechanisms of eosinophilic inflammation are uncertain. Here, it is shown that the nasal secretions of ECRS patients have high eosinophil extracellular trap (EET) and cell‐free DNA (cfDNA) levels. Moreover, the cfDNA induced EET formation by activating toll‐like receptor 9 (TLR9) signaling. After demonstrating that DNase I reduced eosinophilic inflammation by modulating EET formation, linear polyglycerol‐amine (LPG_A_)‐coated TiS_2_ nanosheets (TLPG_A_) as functional 2D nanoplatforms with low cytotoxicity, mild protein adsorption, and increased degradation rate is developed. Due to the more flexible linear architecture, TLPG_A_ exhibited higher cfDNA affinity than the TiS_2_ nanosheets coated with dendritic polyglycerol‐amine (TDPG_A_). TLPG_A_ reduced cfDNA levels in the nasal secretions of ECRS patients while suppressing cfDNA‐induced TLR9 activation and EET formation in vitro. TLPG_A_ displayed exceptional biocompatibility, preferential nasal localization, and potent inflammation modulation in mice with eosinophilic inflammation. These results highlight the pivotal feature of the linear molecular architecture and 2D sheet‐like nanostructure in the development of anti‐inflammation nanoplatforms, which can be exploited for ECRS treatment.

## Graphical abstract

The current therapeutic outcomes of eosinophilic chronic rhinosinusitis (ECRS) remain unsatisfactory. Here, linear polyglycerol‐amine (LPGA)‐covered TiS_2_ nanosheets, which showed low cytotoxicity, mild protein adsorption and robust anti‐inflammation efficacy is developed. These functionalized nanosheets can suppress the eosinophilic extracellular trap (EET) formation and modulate eosinophilic inflammation both in vitro and in vivo.

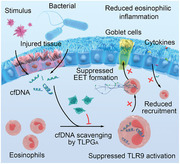

## 1 Introduction

Chronic rhinosinusitis (CRS) is a common nasal inflammatory disease, with symptoms such as nasal obstruction, reduced olfactory function, nasal discharge, and sleep disturbances.^[^ ^1^, ^2^ ^]^ CRS affects over 10% of the global population and is a complex disease, characterized by heterogeneous inflammatory patterns.^[^ ^3^, ^4^ ^]^ CRS is classified into the non‐type 2 or type 2 endotypes, which are associated with mainly neutrophilic and eosinophilic inflammatory patterns, respectively.^[^ ^5^, ^6^ ^]^ Among CRS patients, eosinophilic CRS (ECRS) with type 2 inflammation presents a long‐standing medical challenge for healthcare professionals and researchers alike.^[^ ^6^, ^7^, ^8^ ^]^ This is because patients with ECRS have a more complex etiology, worse prognosis, and increased susceptibility to concomitant asthma recurrence, than those with neutrophilic CRS (NCRS).^[^ ^7^, ^8^ ^]^ Because of the genetic differences between Asian and European populations, neutrophilic inflammation was conventionally more prevalent in Asian CRS patients, whereas eosinophilic inflammation was more common in their European and American counterparts.^[^ ^9^, ^10^ ^]^ However, recent studies have revealed that the proportion of Asian ECRS patients has increased significantly in recent years.^[^ ^9^ ^]^ Moreover, because the pathogenesis of eosinophilic inflammation in ECRS patients is poorly understood, the outcomes of these patients remain suboptimal.^[^ ^10^, ^11^, ^12^ ^]^ Therefore, it is crucial to characterize the dysregulated eosinophilic inflammatory response of ECRS patients, to identify reliable therapeutic targets and develop effective therapeutic strategies.

**Figure 1:**
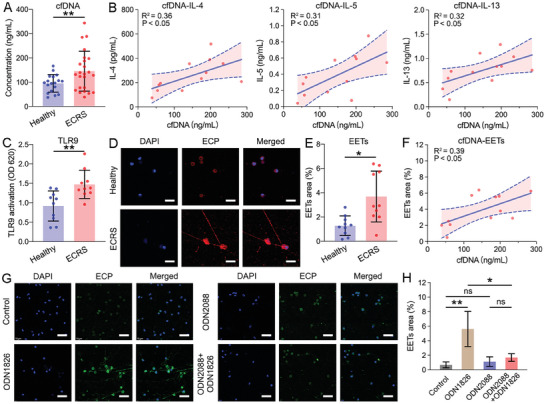
The relationship between cfDNA, EET levels, and eosinophilic inflammation in ECRS patients. A) The cfDNA concentration in nasal secretions from healthy volunteers and ECRS patients. B) The correlation between cfDNA concentration and IL‐4, IL‐5, and IL‐13 levels in the nasal secretions from ECRS patients. C) TLR9 activation induced by the nasal secretions of healthy volunteers and ECRS patients. D) Representative images of DAPI and ECP co‐staining of nasal secretion smears from healthy volunteers and ECRS patients. Scale bars: 20 µm. E) Quantification of the EETs area in the CLSM images of nasal secretions. F) The correlation between cfDNA concentration and the EETs area in the nasal secretions of ECRS patients. G) Representative images of DAPI and ECP co‐staining of eosinophils after incubation with medium only, ODN2088, ODN1826, or ODN2088+ODN1826. Scale bars: 50 µm. H) Quantification of EETs area in the above CLSM images. Data represent the mean ± S.D. (Student's *t*‐test, ns: no significant difference, *p<0.05, **p<0.01).

Cell‐free DNA (cfDNA), which is released from the injured cells of inflamed tissues, was recently revealed as a novel therapeutic target in inflammation‐related diseases, including systemic lupus erythematosus (SLE), rheumatoid arthritis (RA), and inflammatory bowel disease (IBD).^[^ ^13^, ^14^, ^15^, ^16^ ^]^ In the present study, we found that the cfDNA level in the nasal secretions of ECRS patients was significantly higher than that in the nasal secretions of healthy volunteers (**Figure** **1**A); this finding suggested that cfDNA was likely involved in the eosinophilic inflammatory response of ECRS patients. cfDNA is involved in a series of inflammation‐related signaling pathways, such as toll‐like receptors (TLRs), nuclear factor‐kappa B (NF‐κB), interferon regulatory factor (IRF), and mitogen‐activated protein kinase (MAPK), thereby producing a series of pro‐inflammatory cytokines and chemokines.^[^ ^17^, ^18^ ^]^ Specifically, eosinophils play a pivotal role in the eosinophilic inflammatory cascade of ECRS patients.^[^ ^7^, ^8^ ^]^ Similar to the neutrophil extracellular traps (NETs) released by neutrophils, eosinophils can release extracellular chromatin to form eosinophil extracellular traps (EETs) under specific conditions, which result in EET‐mediated cell death (EETosis).^[^ ^19^, ^20^ ^]^ However, EETs have thicker fibers (with globular structures) and are less susceptible to proteolytic degradation than NETs because eosinophils display lower levels of protease activity than neutrophils.^[^ ^19^, ^20^ ^]^ Although EETs protect cells against infection, they may inadvertently cause injury to the surrounding tissues, resulting in diseases such as bronchial asthma, atopic dermatitis, and contact dermatitis.^[^ ^21^, ^22^ ^]^ It was reported that EET formation promotes goblet‐cell hyperplasia, mucus production, infiltration of inflammatory cells, and the expression of type 2 cytokines; collectively, these factors can induce eosinophilic airway inflammation.^[^ ^21^, ^22^ ^]^ Consistent with these previous findings, we detected high EET levels in the nasal secretions of ECRS patients (Figure 1D and E). Because cfDNA was reported to be involved in several inflammatory pathways, including the degranulation of neutrophils and NET formation,^[^ ^23^, ^24^ ^]^ we decided to study whether cfDNA could activate eosinophils and thereby trigger eosinophilic inflammatory pathway in ECRS patients.

Given that cfDNA is involved in many inflammatory diseases, functionalized biomaterials with cfDNA scavenging properties have been successfully applied as novel therapeutic agents to control cfDNA‐associated inflammation.^[^ ^25^, ^26^, ^27^, ^28^ ^]^ We surmised that such a cfDNA scavenging strategy could also potentially attenuate eosinophilic inflammation in ECRS patients. However, the efficacy of current cfDNA scavengers is largely restricted by their unsatisfactory cfDNA binding capacity, non‐specific protein adsorption, low inflammation‐targeting efficacy, and unavoidable biotoxicity.^[^ ^29^, ^30^ ^]^ Fortunately, polyglycerol (PG) and its derivatives offer significant advantages in biomedical applications, including their robust chemical stability, excellent biocompatibility, and reduced protein binding.^[^ ^31^, ^32^ ^]^ Interestingly, linear PG (LPG) and dendritic PG (DPG), which are synthesized both via anionic ring‐opening polymerization, exhibit different physiochemical and biomedical properties due to the linear and dendritic molecular architectures.^[^ ^31^ ^]^ Moreover, the construction of functional 2D nanoplatforms which have unique nanoscale geometry compared to the traditional spherical nanoparticles, represents a promising new approach for therapeutic nanoplatforms design.^[^ ^24^ ^]^ Therefore, it is markedly significant to explore the role of molecular architecture and nanoscale geometry in the development of anti‐inflammation functional nanoplatforms.

In the present study, we showed that cfDNA concentration was elevated in the nasal secretions of ECRS patients, before revealing that cfDNA induced EET formation and elucidating the underlying mechanism. To validate our findings in vivo, we established a mouse model of eosinophilic airway inflammation, in which the inflammatory response was alleviated by treatment with deoxyribonuclease I (DNase I). To avoid the shortcomings of DNase I in clinical application, we constructed LPG_A_‐coated MoS_2_ (MLPG_A_), LPG_A_‐coated TiS_2_ (TLPG_A_), LPG_A_‐coated WS_2_ (WLPG_A_), and DPG_A_‐coated TiS_2_ (TDPG_A_). After comparing the cfDNA binding capacity, biocompatibility, protein adsorption, and stability of these agents, TLPG_A_ was selected as the preferred candidate. Next, we assessed the cfDNA scavenging ability of TLPG_A_ in injured airway epithelial cells and its effect on subsequent EET formation in vitro. In addition, we evaluated the accumulation of TLPG_A_ at inflammatory sites after intranasal administration into mice with eosinophilic inflammation. Finally, we examined the levels of cytokines and EETs, as well as the extent of goblet cell hyperplasia, in the nasal mucosa of model mice after TLPG_A_ administration.

## 2 Results and Discussion

### 2.1 cfDNA and EET Levels in the Nasal Secretions of ECRS Patients

Using expansive sponges, we collected nasal secretions from ECRS patients and healthy volunteers (as negative controls).^[^ ^33^ ^]^ Subsequently, the levels of cfDNA and inflammatory cytokines in the nasal secretions were determined using pico‐green and enzyme‐linked immunosorbent assay (ELISA) kits, respectively.^[^ ^34^, ^35^ ^]^ The results revealed that the cfDNA levels in the nasal secretions of ECRS patients (145.3±82.2 ng mL^−1^) were significantly higher than those in the nasal secretions of healthy volunteers (95.3±36.1 ng mL^−1^) (Figure 1A). Moreover, we found that the cfDNA concentration was closely related to the levels of type 2 inflammatory cytokines IL‐4, IL‐5, and IL‐13 (Figure 1B).^[^ ^36^, ^37^ ^]^ cfDNA is reported to be involved in many inflammatory diseases via TLR9 activation.^[^ ^13^, ^14^, ^15^ ^]^ Consistent with these previous reports, we detected higher (≈150%) levels of TLR9 activation in HEK‐TLR9 cells exposed to the nasal secretions of ECRS patients than to those of healthy volunteers (Figure 1C). The above data indicate that cfDNA was implicated in the inflammatory response of ECRS patients.

As mentioned previously, eosinophil infiltration into the nasal mucosa and polyps is a characteristic of ECRS patients;^[^ ^8^, ^9^, ^10^ ^]^ moreover, once stimulated, these eosinophils could degranulate and form EETs, which is closely related to the eosinophilic inflammatory response.^[^ ^20^, ^21^, ^22^ ^]^ We therefore next prepared nasal secretion smears and performed eosinophil cationic protein (ECP) staining to identify EETs (Figure 1D).^[^ ^21^, ^22^ ^]^ Quantification of the EETs area using confocal laser scanning microscopy (CLSM) revealed increased EET levels in the nasal secretions of ECRS patients than in those of healthy volunteers (Figure 1E). Furthermore, we found a positive correlation (R^2^ = 0.39, P<0.05) between cfDNA concentration and EET levels in the nasal secretions (Figure 1F). These results prompted us to investigate whether EET formation was related to cfDNA level and TLR9 pathway activation.

To delineate the mechanism of EET formation, eosinophils were incubated with ODN1826, a commonly used cfDNA analogue.^[^ ^38^ ^]^ In another experimental group, eosinophils were treated with the TLR9 antagonist ODN2088 for 1 h before ODN1826 was added to the medium (Figure 1G). Quantification of the ECP‐positive area in the CLSM images showed that ODN1826 treatment markedly increased EET formation, while this effect was largely attenuated by pre‐treatment with ODN2088 (Figure 1H). These results indicate that cfDNA triggered EET formation via TLR9 activation, thereby eliciting the eosinophilic inflammatory response in ECRS patients.

### 2.2 DNase I Treatment Reduced Eosinophilic Inflammation in Model Mice

**Figure 2:**
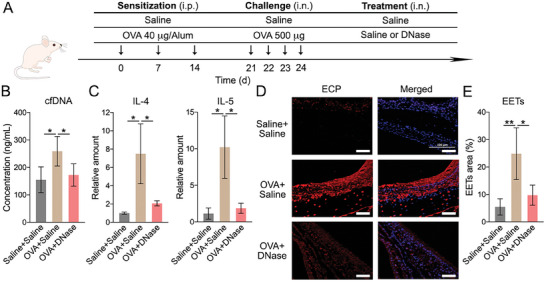
DNase I reduced cfDNA level and alleviated eosinophilic inflammation in a mouse model. A) Schematic diagram of the experimental protocol used to establish the mouse model with eosinophilic airway inflammation and DNase I administration. B) The cfDNA levels in the NALF of experimental mice. C) The relative expression levels of IL‐4 and IL‐5 in the nasal mucosa of experimental mice. D) Representative ECP staining images of the nasal mucosa of experimental mice. Scale bars: 50 µm. E) Quantification of the ECP‐positive area (%) in the immunostaining images. Data represent the mean ± S.D. (*n* = 6, Student's *t*‐test, *p<0.05, **p<0.01).

As cfDNA‐induced EET formation can potentially elicit a long‐lasting uncontrolled eosinophilic inflammatory response in ECRS patients, we used DNase I to investigate whether cfDNA and EETs could be targeted to reduce eosinophilic inflammation.^[^ ^39^, ^40^ ^]^ A mouse model of eosinophilic airway inflammation was set up using ovalbumin (OVA) and Al(OH)_3_ intraperitoneal (i.p.) sensitization on days 0, 7, and 14, according to the published protocol (**Figure** **2**A).^[^ ^41^, ^42^ ^]^ On days 21–24, the mice were intranasally (i.n.) challenged with OVA once per day (four times in total) and then i.n. treated with DNase I at 4 h after each challenge. Mice that were sensitized, challenged, and treated with saline were used as a negative control (NC), while mice that were sensitized and challenged with OVA and then treated with saline were considered as the positive control (PC). Nasal lavage fluids (NALF) were collected at 24 hours after the last treatment. We detected elevated cfDNA concentrations (from 155±47 to 259±54 ng mL^−1^) in the OVA‐sensitized and challenged mice (Figure 2B). Additionally, inflammatory cytokine expression levels in the nasal mucosa of experimental mice were determined by quantitative real‐time polymerase chain reaction (qRT‐PCR). The results confirmed that IL‐4 and IL‐5 expression was elevated in the model mice, implying that eosinophilic nasal inflammation was successfully established in these animals (Figure 2C).^[^ ^41^, ^42^ ^]^ As expected, DNase I treatment significantly reduced the cfDNA concentration (reduced by ≈35%), as well as IL‐4 and IL‐5 expression (decreased by ≈70% and ≈80%) in the nasal mucosa of model mice (Figure 2B and C). These findings indicate that DNase I administration reduced eosinophilic inflammation in vivo. Subsequently, the mice were decapitated, and their maxillary bones (MBs) were collected. The MBs were decalcified in ethylene diamine tetraacetic acid (EDTA) solution for 21 days before being embedded in paraffin and sectioned.^[^ ^43^ ^]^ Finally, the tissue slices were subjected to ECP immunostaining^[^ ^21^, ^22^ ^]^ and we observed higher EET formation in OVA‐treated mice than in untreated mice (Figure 2D and E). In line with the cfDNA and cytokine levels, the amount of EETs in the nasal mucosa of mice with eosinophilic inflammation was also decreased (from 24.9±9.4% to 9.7±3.7%) after DNase I treatment (Figure 2D and E). Although DNase I treatment markedly reduced eosinophilic inflammation, its clinical application is severely limited by low stability and side effects, such as voice changes and rash.^[^ ^39^ ^]^ Therefore, developing biomaterials‐based cfDNA scavenging nanoplatforms may be an alternative promising therapeutic strategy for dampening the inflammatory response of ECRS patients.

### 2.3 Preparation of LPG_A_‐Coated and DPG_A_‐Coated 2D Nanoplatforms

Because cfDNA is negatively charged owing to the presence of anionic phosphates in the DNA strands, several cationic polymers, such as polyethyleneimine (PEI) and polyamidoamine (PAMAM), have been developed as cfDNA scavengers.^[^ ^13^, ^14^, ^15^, ^16^, ^25^, ^26^, ^27^, ^28^ ^]^ However, these polymers display either unsatisfactory cfDNA binding efficacy or excessive toxicity, which restricts their translation into the clinic. In addition, the protein adsorption property of cationic polymers suppresses their cfDNA scavenging efficacy, especially in vivo.^[^ ^29^, ^30^ ^]^ In recent decades, PG, including LPG and DPG, which are polymers exhibiting low levels of toxicity and few intrinsic interactions with proteins, was developed for biomedical applications.^[^ ^31^, ^44^ ^]^ In addition, some of the hydroxyls of LPG and DPG molecules can be converted to amino groups to obtain LPG‐amine (LPG_A_) and DPG‐amine (DPG_A_), which further promotes cfDNA binding by strengthening electrostatic forces.^[^ ^44^ ^]^ The synthesis and characterization of LPG_A_ and DPG_A_ are outlined in the Figures S1A and S2A (Supporting Information). Although cationic polymer‐based nanoplatforms could be applied as anti‐inflammation cfDNA scavengers,^[^ ^27^, ^28^ ^]^ it is still unclear if the molecular architecture could affect their cfDNA binding capacity as well as the anti‐inflammation effect. Due to the corresponding linear and dendritic molecular architectures, LPG and DPG displayed distinct physiochemical and biomedical properties.^[^ ^45^ ^]^ Moreover, 2D nanoplatforms with sheet‐like nanostructures always exhibit unique biological characteristics compared to spherical nanoparticles.^[^ ^46^ ^]^ Therefore, we explored the role of molecular architecture and nanoscale geometry in cfDNA scavengers’ development for inflammation modulation.

In this study, we selected several transition‐metal dichalcogenides, including molybdenum disulfide (MoS_2_), titanium disulfide (TiS_2_), and tungsten disulfide (WS_2_), to comprehensively investigate the role of 2D morphology in cfDNA scavenger development.^[^ ^46^, ^47^ ^]^ MoS_2_, TiS_2,_ and WS_2_ nanosheets were prepared via lithium‐ion intercalation and then modified using lipoic acid (LA).^[^ ^47^ ^]^ Afterward, the nanosheets were coated with LPG_A_ using an amidation reaction to obtain LPG_A_‐coated MoS_2_ (MLPG_A_), TiS_2_ (TLPG_A_), and WS_2_ (WLPG_A_) (Figures S1B and S2B, Supporting Information).^[^ ^47^ ^]^ Next, the composition of MLPG_A_, TLPG_A,_ and WLPG_A_ was assessed and the percentage of LPG_A_ in MLPG_A_, TLPG_A_, and WLPG_A_ was calculated (81.8%, 80.2%, and 76.7%, respectively) by UV adsorption at 360 nm (Figures S5–S8, Supporting Information). TiS_2_ covered with DPG_A_ (TDPG_A_) was also prepared to investigate the effect of linear/dendritic molecular architecture on the construction of cfDNA scavengers.

**Figure 3:**
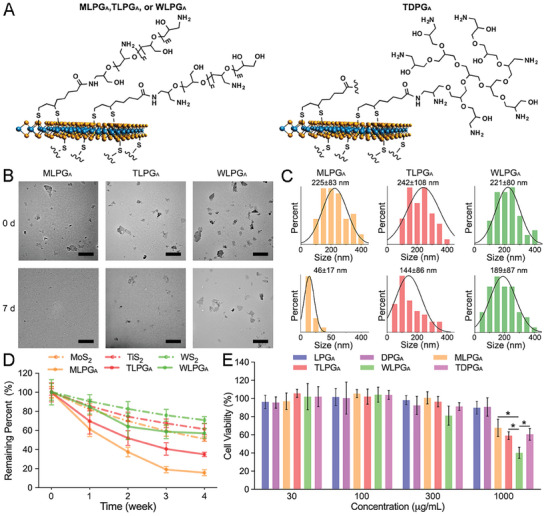
Characterizations of functional 2D nanoplatforms. A) The composition and molecular architecture of MLPG_A_, TLPG_A_, WLPG_A_ and TDPG_A_. B) Representative TEM images of MLPG_A_, TLPG_A,_ and WLPG_A_ before and after incubation in PBS (pH 7.4) at 37 °C for 7 days. Scale bars: 500 nm. C) Size profiles of MLPG_A_, TLPG_A_, and WLPG_A_ before and after incubation in PBS (pH 7.4) at 37 °C for 7 days (based on the TEM images). D) Relative absorbance (360 nm) of MoS_2_, TiS_2_, WS_2_, MLPG_A_, TLPG_A,_ and WLPG_A_ during a 4‐week incubation in PBS (pH 7.4). E) Viability of BEAS‐2B cells treated for 24 h with various concentrations of LPG_A_, DPG_A_, MLPG_A_, TLPG_A_, WLPG_A_, and TDPG_A_. Data represent the mean ± S.D. (n = 3, Student's *t*‐test, *p<0.05, **p<0.01).

We next used transmission electron microscopy (TEM) to show that although MLPG_A_, TLPG_A_, WLPG_A,_ and TDPG_A_ differed in their composition, their 2D sheet‐like nanostructures were similar (**Figure** **3**B; Figure S9, Supporting Information). The size distributions of MLPG_A_, TLPG_A,_ WLPG_A_, and TDPG_A_ were 225±83, 242±108, 221±80, and 218±67 nm, respectively (Figure 3C; Figure S9, Supporting Information). The extent of biodegradability, which is an important parameter in the development of biomaterials, was assessed using TEM images and UV adsorption (360 nm) data.^[^ ^48^ ^]^ We found that after incubation in PBS (pH 7.4) at 37 °C for 7 days, the degradation rates of MLPG_A_, TLPG_A_, and TDPG_A_ were markedly higher than that of WLPG_A_ (Figure 3; Figure S9, Supporting Information). Subsequently, we found the cytotoxicity of MLPG_A_, TLPG_A_, and TDPG_A_ nanosheets was significantly lower than that of WLPG_A_ (Figure 3E; Figure S10, Supporting Information). These findings indicated that MLPG_A_, TLPG_A_, and TDPG_A_ had higher levels of biosafety and were more suitable for in vivo application.

### 2.4 cfDNA Scavenging by Functional 2D Nanoplatforms

**Figure 4:**
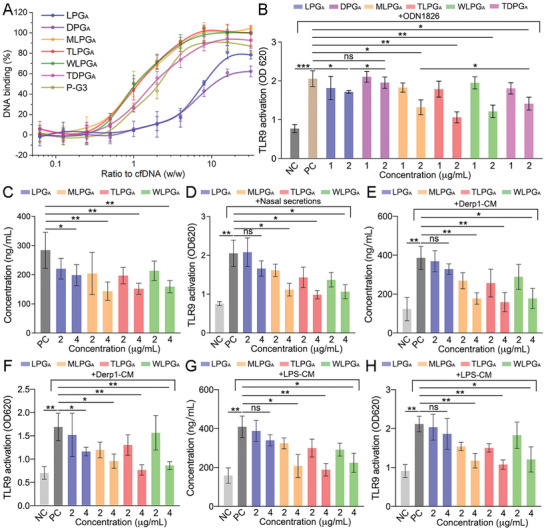
MLPG_A_, TLPG_A,_ and WLPG_A_ suppressed TLR9 activation via cfDNA scavenging. A) cfDNA binding efficiency of LPG_A_, DPG_A_, MLPG_A_, TLPG_A_, WLPG_A_, TDPG_A_, and P‐G3 in FBS (10%) solutions. B) TLR9 activation of HEK‐TLR9 cells induced by ODN1826+LPG_A_, ODN1826+DPG_A_, ODN1826+MLPG_A_, ODN1826+TLPG_A_, ODN1826+WLPG_A_, or ODN1826+TDPG_A_. C) cfDNA levels in nasal secretions from ECRS patients after incubation with LPG_A_, MLPG_A_, TLPG_A_, or WLPG_A_. D) TLR9 activation of HEK‐TLR9 cells treated with nasal secretions from ECRS patients after incubation with LPG_A_, MLPG_A_, TLPG_A_, or WLPG_A_. E) cfDNA levels in Derp1‐CM after incubation with LPG_A_, MLPG_A_, TLPG_A_, or WLPG_A_. F) TLR9 activation of HEK‐TLR9 cells induced by Derp1‐CM after incubation with LPG_A_, MLPG_A_, TLPG_A_, or WLPG_A_. G) cfDNA levels in LPS‐CM after incubation with LPG_A_, MLPG_A_, TLPG_A_, or WLPG_A_. H) TLR9 activation of HEK‐TLR9 cells induced by LPS‐CM after incubation with LPG_A_, MLPG_A_, TLPG_A_, or WLPG_A_. For the cfDNA release experiments, BEAS‐2B cells incubated with medium only were considered as a negative control (NC) and BEAS‐2B cells incubated with Derp1 only or LPS only were considered as a positive control (PC). For the TLR9 activation experiments, HEK‐TLR9 cells incubated with medium only were considered as a NC and HEK‐TLR9 cells incubated with ODN1826 only, nasal secretions only, or CM only were considered as a PC. Data represent the mean ± S.D. (*n* = 3, Student's *t*‐test, ns: no significant difference, *p<0.05, **p<0.01, ***p<0.001).

As the functional 2D nanoplatforms were designed to scavenge cfDNA via electrostatic forces, we next measured the zeta potential of MLPG_A_, TLPG_A_, WLPG_A,_ and TDPG_A_ and obtained 13.9±1.8, 14.8±1.8, 15.1±2.8, and 15.5±1.9 mV, respectively (Figure S11, Supporting Information). The stability of functional 2D nanoplatforms in a physiological‐like environment was also confirmed (Figures S11 and S12, Supporting Information). We then quantified the extent of cfDNA binding in pure water and found that LPG_A_ displayed higher binding efficacy than DPG_A_ and similar results were also observed with TLPG_A_ and TDPG_A_ (**Figure** **4**A; Figure S13, Supporting Information). These results indicated the superiority of linear molecular architecture over dendritic molecular architecture for cfDNA binding, which was attributed to the higher flexibility as well as stronger multivalent interaction.^[^ ^45^ ^]^ MLPG_A_, TLPG_A,_ and WLPG_A_ displayed cfDNA binding efficacies that were similar to that of PAMAM generation 3 (P‐G3) but considerably higher than that of LPG_A_, indicating the importance of the 2D nanostructure in the development of anti‐inflammation nanoplatforms (Figure S13, Supporting Information). However, while the cfDNA scavenging by P‐G3 decreased considerably in solutions containing fetal bovine serum (FBS) (10%), the LPG_A_‐coated nanoplatforms retained their robust cfDNA binding efficacy (Figure 4A; Figure S14, Supporting Information); this quality can be attributed to the protein resistant properties of PG‐based nanomaterials (Figure S15, Supporting Information).^[^ ^49^ ^]^ As previously mentioned, TLR9 activation is crucial for EET formation by eosinophils. We, therefore, wondered whether the LPG_A_‐coated nanosheets could inhibit cfDNA‐induced TLR9 activation. We treated HEK‐TLR9 cells (HEK293 cells gene‐edited to express TLR9) with the cfDNA analogue ODN1826 and measured their level of TLR9 activation. The results showed that MLPG_A_, TLPG_A,_ WLPG_A_, and TDPG_A_ (2 µg mL^−1^) effectively suppressed ODN1826‐induced TLR9 activation. Moreover, these functional 2D nanoplatforms were more effective than LPG_A_ and DPG_A_ alone and TLPG_A_ exhibited higher TLR9 inhibition than TDPG_A_ (Figure 4B), confirming the advantages of 2D sheet‐like geometry and linear molecular architecture of functional nanoplatforms for cfDNA scavenging. Collectively, these findings demonstrate that LPG_A_‐coated nanosheets could effectively bind to cfDNA and suppress cfDNA‐induced TLR9 pathway activation, suggesting that these functional 2D nanoplatforms with linear architecture could potentially be used as anti‐inflammatory agents to treat ECRS. Therefore, MLPG_A_, TLPG_A_, and WLPG_A_ were selected for the following studies.

We next showed that treatment with MLPG_A_, TLPG_A,_ or WLPG_A_ reduced the cfDNA levels in the nasal secretions of ECRS patients; again, these nanosheets were more effective at reducing cfDNA levels than LPG_A_ alone, especially when used at 4 µg mL^−1^ (Figure 4C). In accordance, the LPG_A_‐coated nanosheets also effectively reduced TLR9 activation induced by the nasal secretions of ECRS patients (Figure 4D). Exposure to stimulus and bacterial infection are the main triggers of ECRS.^[^ ^6^, ^7^, ^8^ ^]^ Thus, we next treated BEAS‐2B cells with Derp1 and lipopolysaccharide (LPS), derived from *Dermatophagoides pteronyssinus* and Gram‐negative bacteria, respectively.^[^ ^6^, ^7^, ^8^ ^]^ We observed that the cfDNA level in the cell culture medium increased from < 200 to ≈400 ng mL^−1^ after incubation with Derp1 (Figure 4E). Moreover, the Derp1‐conditioned medium (Derp1‐CM) induced a 3‐fold increase in TLR9 activation (Figure 4F). In line with our previous results, treatment with MLPG_A_, TLPG_A,_ or WLPG_A_ (4 µg mL^−1^) reduced the cfDNA concentration and TLR9 activation to normal levels; meanwhile, LPG_A_ exerted a much weaker effect. Similar to Derp1 and Derp1‐CM, LPS‐induced cfDNA release and LPS‐conditioned medium (LPS‐CM)‐triggered TLR9 activation were also observed (Figure 4G and H). These findings indicate that cfDNA was released from injured epithelial cells after exposure to stimulus or infection, thereby triggering an inflammatory cascade. Moreover, the LPG_A_‐coated nanosheets exhibited higher cfDNA scavenging ability and TLR9 inhibition than LPG_A_, reconfirming the significant role of the 2D nanostructure in the construction of cfDNA scavenging nanoplatforms. Although MLPG_A_, TLPG_A,_ and WLPG_A_ exhibited similar cfDNA scavenging efficacies, MLPG_A_ and TLPG_A_ were degraded faster than WLPG_A_, meaning that they had higher levels of biosafety. In addition, the degradation product of TLPG_A_ is mainly TiO_2,_ which is a non‐toxic compound widely used as an additive in the food and cosmetics industries.^[^ ^50^ ^]^ Thus, TLPG_A_ was selected for subsequent anti‐inflammation studies and LPG_A_ was used as a control.

### 2.5 cfDNA‐Induced EET Formation was Suppressed by TLPG_A_

**Figure 5:**
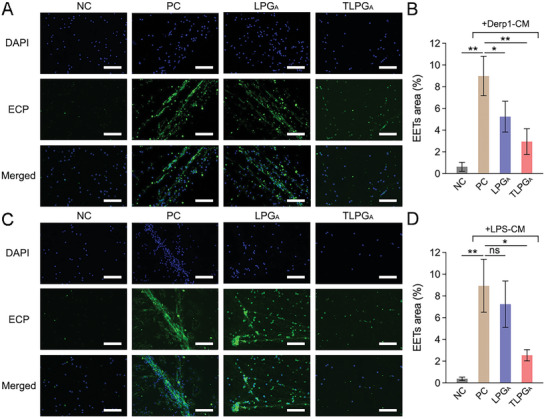
TLPG_A_ reduced EET formation in vitro. A) Representative DAPI and ECP co‐staining images of the Derp1‐CM‐treated eosinophils after incubation with LPG_A_ or TLPG_A_. Scale bars: 100 µm. B) Quantification of the EETs area in the representative CLSM images. C) Representative DAPI and ECP co‐staining images of the LPS‐CM‐treated eosinophils after incubation with LPG_A_ or TLPG_A_. Scale bars: 100 µm. D) Quantification of the EETs area in the representative CLSM images. Eosinophils incubated with medium‐only or CM‐only were considered as the NC or PC, respectively. Data represent the mean ± S.D. (*n* = 3, Student's *t*‐test, ns: no significant difference, *p<0.05, **p<0.01).

Given that TLPG_A_ could efficiently suppress cfDNA‐induced TLR9 activation, which is related to EET formation, we next examined whether TLPG_A_ could reduce EET formation via cfDNA scavenging. To this end, we pre‐treated BEAS‐2B cells with Derp1 or LPS for 12 h and then replaced the medium with fresh medium to avoid the influence of the original stimulus in the subsequent experiments. After another 12 h incubation, the Derp1‐CM and LPS‐CM were collected. Subsequently, we cultured eosinophils isolated from the peripheral blood of ECRS patients for 4 h before replacing the medium with Derp1‐CM or LPS‐CM containing LPG_A_ or TLPG_A_. EET formation was determined by ECP immunostaining after a 24 h incubation and the representative CLSM images were collected for quantification (**Figure** **5**A). The results showed that Derp1‐CM induced EET formation, as evidenced by the increase in the EETs area within the CLSM images from 0.62±0.42% to 8.99±1.82% (Figure 5B). Consistent with the cfDNA scavenging results, the Derp1‐CM‐induced EET formation was suppressed by TLPG_A_ to 2.95±1.18% (Figure 5B). Similar results were observed in the LPS‐CM‐treated eosinophils, whereby TLPG_A_ administration decreased the EETs area from 8.93±2.43% to 2.54±0.51% (Figure 5C and D). In comparison, LPG_A_ did not have a significant effect on EET formation (Figure 5C and D). Thus, our data revealed that TLPG_A_ efficiently blocked TLR9 stimulation as well as EET formation via cfDNA scavenging.

### 2.6 Nasal Accumulation and Biodistribution of LPG_A_ and TLPG_A_

**Figure 6:**
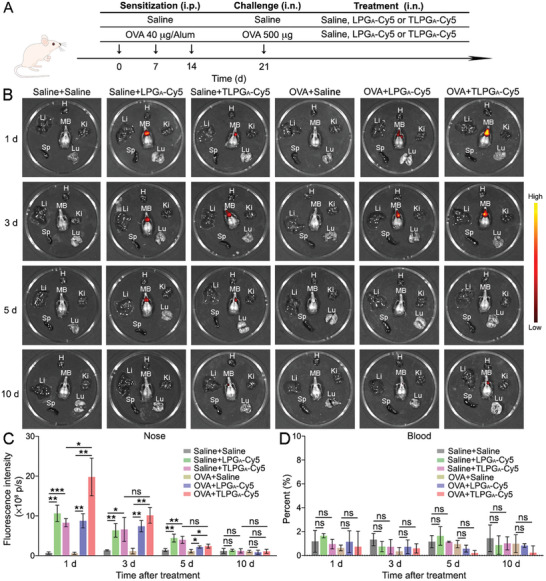
Biodistribution of LPG_A_‐Cy5 and TLPG_A_‐Cy5 after intranasal treatment. A) A schematic diagram outlining the experimental protocol used to establish and treat mice with eosinophilic inflammation. B) Ex vivo fluorescence imaging of major organs and MBs of the experimental mice at different time points post‐treatment. H: heart; Li: liver; S: spleen; Lu: lung; K: kidney; MB: maxillary bone. C) Quantification of the fluorescence intensity in the noses of experimental mice. D) Quantification of nanomaterial concentration in the serum of experimental mice on the basis of fluorescence intensity. Data represent the mean ± S.D. (*n* = 3, Student's *t*‐test, ns: no significant difference, *p<0.05, **p<0.01, ***p<0.001).

As TLPG_A_ potently suppressed cfDNA‐induced EET formation in vitro, we next wanted to examine its therapeutic effect in vivo using a mouse model of eosinophilic inflammation. However, prior to this investigation, we first assessed the biodistribution and accumulation of LPG_A_ and TLPG_A_ in the nasal tissues of sham mice and model mice with eosinophilic inflammation (**Figure** **6**A).^[^ ^51^ ^]^ Thus, Cy5‐labeled LPG_A_ (LPG_A_‐Cy5) or TLPG_A_ (TLPG_A_‐Cy5) were i.n. instilled into the mice and their biodistribution in the nose and other major organs was recorded on day 1, 3, 5, and 10 post‐treatment (Figure 6B).^[^ ^52^ ^]^ The fluorescent signals originating from noses peaked on day 1 post‐treatment but decreased quickly thereafter (Figure 6B and C). The mice were then sacrificed and their MBs, as well as other major organs (heart, liver, spleen, lungs, and kidneys), were isolated. The ex vivo fluorescence images showed that the fluorescent signals were strongest in the nose, which is crucial for rhinosinusitis treatment (Figure 6B and C). Of note, a 2‐fold greater accumulation of TLPG_A_ was observed in the noses of model mice than in those of sham mice, which suggests that TLPG_A_ preferentially localized to sites of inflammation (Figure 6C). This inflammation‐targeting property of TLPG_A_ is not only likely to increase its therapeutic effect in inflamed tissues but also reduce side effects in healthy tissues.^[^ ^53^ ^]^ The fluorescent signals detected in serum were applied to investigate the quantity of nanomaterials in blood. Indeed, we confirmed that <2% of the nanomaterials were circulating in the blood after treatment, confirming the inflammatory targeting properties of LPG_A_ and TLPG_A_ (Figure 6D). Moreover, the fluorescent signals were barely detectable (not significant compared with NC group) in the hearts, livers, spleens, and kidneys (Figure 6B; Figure S17, Supporting Information), confirming the biosafety of LPG_A_ and TLPG_A_ during intranasal treatment. Importantly, the fluorescent signals in the noses of mice were significantly reduced by day 5 and were almost undetectable at day 10 post‐treatment (Figure 6B and C); this was due to the biodegradation of the nanomaterials and their eventual excretion from the nasal cavity.^[^ ^48^ ^]^

### 2.7 TLPG_A_ Alleviated Eosinophilic Inflammation In Vivo

**Figure 7:**
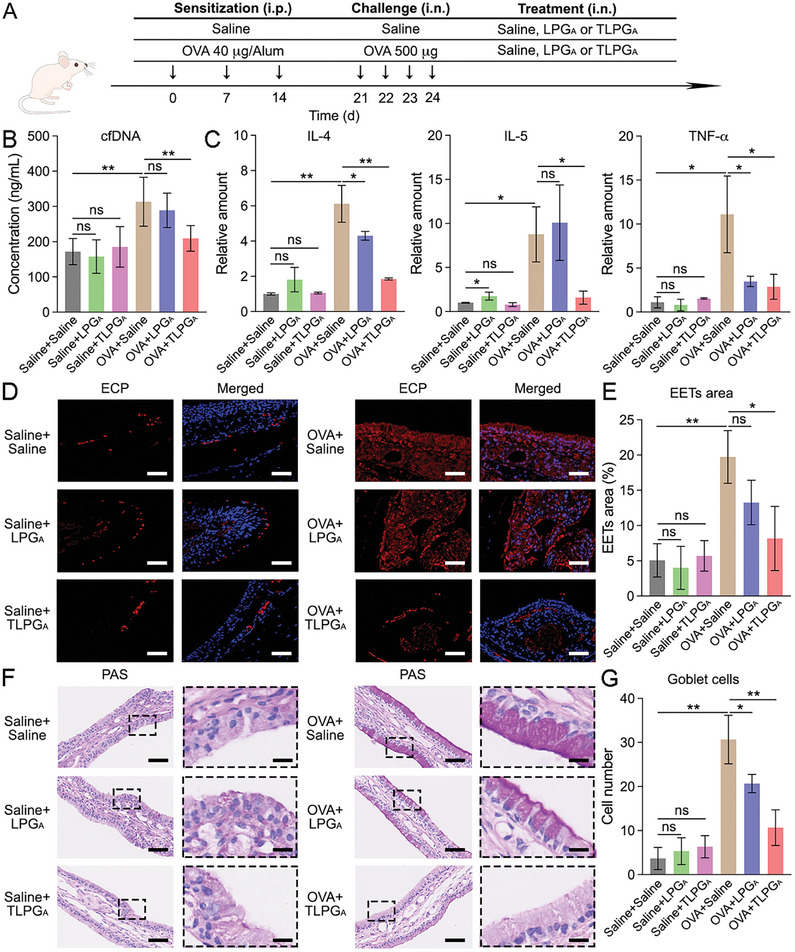
TLPG_A_ attenuated eosinophilic inflammation in vivo via cfDNA scavenging. A) A schematic diagram outlining the experimental protocol of TLPG_A_ administration to mice with eosinophilic inflammation. B) The cfDNA levels in the NALF of experimental mice in the different treatment groups. C) The relative expression levels of IL‐4, IL‐5, and TNF‐α in the nasal mucosa of the experimental mice. D) Representative ECP immunostaining images of the nasal mucosa of the experimental mice. Scale bars: 50 µm. E) Quantification of the ECP‐positive area (%) in the immunostaining images. F) Representative PAS staining images of the nasal mucosa of the experimental mice. Scale bars in the low magnification images: 50 µm; scale bars in the amplified sections: 12.5 µm. G) Quantification of goblet cells in the PAS staining images. Data represent the mean ± S.D. (*n* = 6, Student's *t*‐test, ns represents no significant difference, *p<0.05, **p<0.01, ***p<0.001).

Having shown that TLPG_A_ preferentially accumulated in the inflamed tissues of OVA‐treated model mice, we next examined its effect on OVA‐induced inflammation in more detail (**Figure** **7**A). First, we showed that neither LPG_A_ nor TLPG_A_ treatment increased cfDNA levels in the NALF of the experimental mice (Figure 7B). In addition, the qRT‐PCR results showed that neither LPG_A_ nor TLPG_A_ significantly increased the expression of inflammatory cytokines (i.e., IL‐4, IL‐5, IL‐6, IL‐17, TNF‐α, and IFN‐γ) in the nasal mucosa of the treated mice (Figure 7C; Figure S18, Supporting Information), confirming the biosafety of LPG_A_‐based nanomaterials for in vivo application. Next, a mouse model of eosinophilic airway inflammation was set up by OVA sensitization and challenge, as previously described.^[^ ^41^, ^42^ ^]^ Interestingly, the results demonstrated that TLPG_A_ treatment effectively reduced the cfDNA level (from 313±69 to 209±36 ng mL^−1^) in the NALF of mice with inflammation while this cfDNA scavenging effect was much weaker in the LPG_A_‐treated group (Figure 7B). Subsequently, the experimental mice were sacrificed and their nasal mucosal tissues were collected for quantification of inflammatory cytokines expression with qRT‐PCR.^[^ ^54^ ^]^ TLPG_A_ treatment effectively reduced the expression of inflammatory cytokines, especially IL‐4 (by ≈70%) and IL‐5 (by ≈80%), in the nasal mucosa of mice with inflammation (Figure 7C; Figure S21, Supporting Information). Although LPG_A_ treatment slightly reduced the expression of these cytokines (e.g., IL‐4 reduced by ≈30%), its efficacy was much lower than that of TLPG_A_. In addition to quantifying the mRNA expression of cytokines, the effect of LPG_A_ and TLPG_A_ treatment on cytokine expression in the NALF of mice was confirmed by ELISA (Figure S20, Supporting Information).

Because eosinophils and EETs participate in the inflammatory response of ECRS patients, we next set out to characterize eosinophils and EETs in the nasal mucosa of experimental and sham mice. To this end, we collected mouse MBs, which were subsequently decalcified, embedded, sectioned, and immunostained. According to the CLSM images and the corresponding qualification results, LPG_A_ or TLPG_A_ did not promote eosinophil recruitment (Figure S22, Supporting Information) or EET formation (Figure 7D and E), indicating that the treatment would not damage healthy tissues. Consistent with the reduction in eosinophil recruitment, the EET formation in the inflamed tissues was also suppressed by TLPG_A_ treatment. The ECP‐positive areas in the nasal mucosa of sham mice and OVA‐treated mice were 5.1±2.4% and 19.7±3.7%, respectively, indicating that eosinophilic inflammation was induced after OVA sensitization and challenge. TLPG_A_ treatment decreased the ECP‐positive area to 8.2±4.6%, while this value was 13.3±3.2% for the LPG_A_‐treated group (Figure 7D and E). Collectively, these results suggest that the LPG_A_‐coated 2D nanoplatforms efficiently suppressed eosinophil accumulation and EET formation in vivo via cfDNA scavenging.

**Figure 8:**
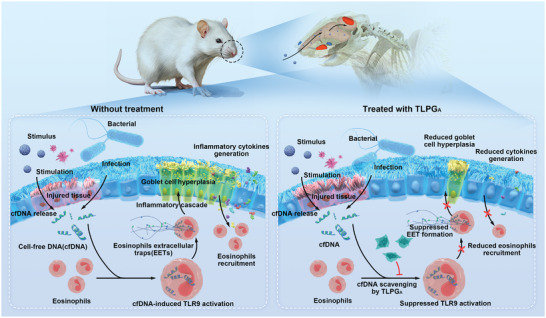
A schematic diagram providing an overview of the dysregulated eosinophilic inflammation of the nasal mucosa modulated by TLPG_A_ nanosheets in model mice.

Goblet cell hyperplasia, which is another characteristic of ECRS, induces excessive mucus secretion in the nasal cavity.^[^ ^55^ ^]^ To investigate the effect of the LPG_A_‐coated nanosheets on this phenomenon, the MBs of experimental mice were stained with periodic acid Schiff (PAS) to identify the goblet cells in the nasal mucosa (Figure 7F and G).^[^ ^55^ ^]^ We found that the mice with eosinophilic inflammation had established goblet cell hyperplasia. TLPG_A_ treatment did not increase the number of goblet cells in nasal mucosa of sham mice, and it reduced the percentage of goblet cells by ≈65% in the OVA‐treated group. By contrast, LPG_A_ only reduced the percentage of goblet cells by ≈30%, indicating that the 2D nanostructure significantly increased the efficacy of this i.n. nanomedicine. Collectively, our results suggest that TLPG_A_ effectively reduced eosinophilic nasal inflammation via cfDNA scavenging and has a promising potential in ECRS management (**Figure** **8**).

In addition to nasal inflammation, i.n. OVA‐challenge induces an inflammatory response in the lungs of experimental mice. To investigate this in more detail, we collected the bronchoalveolar lavage fluid (BALF) of the experimental mice and subjected it to an ELISA.^[^ ^56^ ^]^ The results revealed that TLPG_A_ but not LPG_A_ treatment significantly reduced the production of OVA‐induced pro‐inflammatory cytokines in the lungs of model mice (Figure S23, Supporting Information). In addition, the inflammatory score, calculated from the images of H&E‐stained lung sections, decreased from 3.3±0.8 to 2.0±0.9 after TLPG_A_ treatment (Figure S24, Supporting Information).^[^ ^56^ ^]^ Excessive EET formation was also observed in the lungs of mice with eosinophilic airway inflammation versus those of sham mice (22.3±4.0% and 6.2±1.3%); moreover, TLPG_A_ treatment reduced the EET area in the lungs more effectively than LPG_A_ administration (11.6±2.9% and 15.2±1.4%, respectively) (Figure S25, Supporting Information). Although TLPG_A_ reduced inflammation in the lungs of model mice, its effect was much lower than that observed in the nasal cavity; this difference may be due to the relatively low accumulation of nanomaterials in the lungs after i.n. administration.

### 2.8 Biosafety Evaluation of LPG_A_ and TLPG_A_

**Figure 9:**
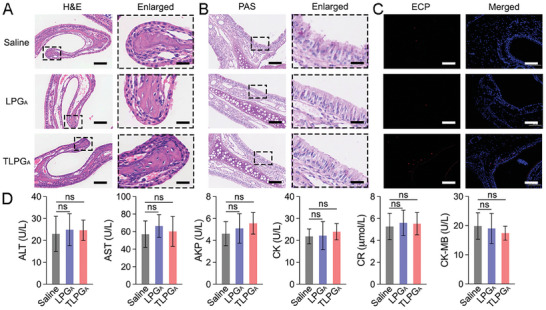
Biocompatibility of LPG_A_ and TLPG_A_. A) Representative H&E staining images of the nasal mucosa of experimental mice on day 14 post‐treatment. Scale bars: 100 µm; scale bars in amplified sections: 25 µm. B) Representative PAS staining images of the nasal mucosa of experimental mice on day 14 post‐treatment. Scale bars: 100 µm; scale bars in amplified sections: 25 µm. C) Representative ECP immunostaining images of the nasal mucosa of experimental mice on day 14 post‐treatment. Scale bars: 100 µm. D) Biochemical parameters, including ALT, AST, AKP, CK, and CR in the serum of experimental mice on day 14 post‐treatment.

Finally, the long‐term safety profiles of LPG_A_ and TLPG_A_ were carefully evaluated at 14 and 28 days in healthy mice after their i.n. administration. Briefly, the mice were i.n. instilled with LPG_A_ or TLPG_A_, before their MBs were isolated and decalcified in EDTA for 14 days. Next, H&E staining, PAS staining, and ECP immunostaining were performed to investigate whether the nanomaterials caused tissue injury. The H&E staining images revealed compact, healthy nasal tissue after LPG_A_ and TLPG_A_ treatment.^[^ ^57^ ^]^ Meanwhile, the PAS staining images showed almost no evidence of goblet cell hyperplasia following LPG_A_ and TLPG_A_ administration, indicating that LPG_A_ and TLPG_A_ exhibited high levels of biosafety (**Figure** **9**A,B). Furthermore, LPG_A_ and TLPG_A_ treatment did not increase EET formation in the nasal mucosa, indicating that these agents were not pro‐inflammatory (Figure 9C). In addition, we observed no obvious signs of injury in the tissues of major organs (including the heart, liver, spleen, lungs, and kidneys) at 14 and 28 days after LPG_A_ or TLPG_A_ treatment (Figures S26 and S27, Supporting Information). The serum of the experimental mice was also collected and the levels of alanine aminotransferase (ALT), aspartate aminotransferase (AST), alkaline phosphatase (AKP), creatine kinase (CK), creatinine (CR), along with CK‐MB, were determined.^[^ ^58^ ^]^ No significant elevation of these indicators was detected, indicating that the hepatic and renal functions of the experimental mice were not impaired following LPG_A_ or TLPG_A_ administration (Figure 9D; Figure S28, Supporting Information). Collectively, the above results provide strong support for the biosafety of LPG_A_‐based nanomaterials, paving the way for their potential clinical application in the treatment of nasal diseases.

## 3 Conclusion

As a distinct endotype of CRS, ECRS is characterized by the infiltration of eosinophils into the nasal cavity and paranasal sinuses, which has a substantial impact on a patient's quality of life and contributes to significant healthcare expenditures. Moreover, the incidence of ECRS is rapidly increasing, while the therapeutic efficacy of the widely used glucocorticoids remains unsatisfactory, highlighting the need for more effective therapies to manage this condition. Previous studies have reported that cfDNA is associated with many inflammatory diseases, including sepsis, inflammatory bowel disease, and rheumatoid arthritis. Consistent with the previous reports, we showed that cfDNA concentrations were elevated in the nasal secretions of ECRS patients (versus healthy volunteers), and were positively correlated with EETs abundance. Subsequently, we confirmed the EET formation was related to cfDNA‐induced TLR9 pathway activation in eosinophils. In addition, we showed that cfDNA degradation by DNase I mitigated eosinophilic inflammation in a mouse model; however, because its clinical application is restricted by low stability and side effects, we developed an alternative cfDNA scavenging strategy using functional nanoplatforms. Four types of LPG_A_‐coated and DPG_A_‐coated nanosheets were prepared, using MoS_2_, TiS_2_, or WS_2_ as the backbone. Fascinatingly, LPG_A_‐covered nanosheets surpassed the cfDNA binding efficacy achieved by the corresponding LPG_A_ polymer; this is likely because the flat, planar structure of the functional 2D nanoplatforms covered with LPG_A_ allows for a better access to the cfDNA molecules. In addition, MLPG_A_, TLPG_A_, and WLPG_A_ displayed stronger electrostatic interaction with cfDNA than TDPG_A_ due to the higher flexibility of linear molecular architecture and 2D nanoscale geometry. Eventually, TLPG_A_ was selected as the preferred candidate because of its higher degradation rate and low cytotoxicity. We demonstrated that TLPG_A_ suppressed cfDNA‐induced TLR9 activation and EET formation more effectively than LPG_A_ alone. Notably, TLPG_A_ preferentially accumulated in the inflamed nasal tissues after intranasal instillation, where it reduced eosinophil recruitment, EET formation, and goblet cell hyperplasia, as well as attenuating the subsequent eosinophilic inflammatory cascade. Therefore, our findings highlight the importance of the linear molecular architecture and 2D nanostructure in the development of cfDNA scavengers and provide a novel treatment strategy for the management of recalcitrant ECRS.

## 4 Experimental Section

### Preparation of MoS_2_, TiS_2_, and WS_2_ Nanosheets

MoS_2,_ TiS_2_, or WS_2_ monolayers were produced according to the publications by using lithium anions as intercalation agents.^[^ ^59^ ^]^ Under argon, 2 g MoS_2,_ TiS_2_, or WS_2_ powder was added to a flask, and 25 mL of n‐butyllithium in n‐hexane was subsequently added. The mixture was condensed and refluxed at 60 °C for 2 d before the reaction solution was centrifuged at 2000 rpm for 5 min. In the next step, the supernatant was discarded, and the precipitate was dispersed in n‐hexane and washed by centrifugation at 2000 rpm twice. Finally, the precipitate was dispersed in Milli‐Q water and dialyzed in Milli‐Q water (MWCO = 12–14 K) for 3 d. MoS_2,_ TiS_2_, or WS_2_ monolayer nanosheets were obtained after lyophilization.

### Synthesis of MLPG_A_, TLPG_A_, and WLPG_A_

Ethoxyethyl glycidyl ether (EEGE) was synthesized from glycidol and ethyl vinyl ether (EVE) and was purified by stirring over CaH_2_.^[^ ^45^ ^]^ LPG (Mw≈5000 g mol^−1^) was synthesized by ring‐opening polymerization and LPG_A_ was synthesized through azidation and reduction reaction according to the literature.^[^ ^45^ ^]^

1 mg mL^−1^ MoS_2_, TiS_2_, or WS_2_ nanosheets in aqueous solution and 10 mg mL^−1^ lipoic acid (LA) solution in methanol were prepared. After that, 0.5 mL LA solution was added to 10 mL MoS_2_, TiS_2_, or WS_2_ solution followed by a reaction at room temperature for 24 h to prepare LA‐modified MoS_2_ (M_LA_), LA‐modified TiS_2_ (T_LA_) and LA‐modified WS_2_ (W_LA_). Then, 10 mg M_LA_, T_LA_, or W_LA_ was dispersed in EDC (80 mg) solution in MES (0.2 M), and the solution was then added dropwise to 1 g LPG in MES (0.2 M). The reaction mixture was stirred at room temperature for 2 d before dialysis in Mili‐Q water (MWCO = 12–14 K) for 2 d to obtain MLPG, TLPG, or WLPG.

100 mg MLPG, TLPG, or WLPG was dissolved in DMF and stirred at room temperature for 1 h. Then MsCl (50 µL) and TEA (0.41 mL) were added to the solution and the mixture was stirred under an argon atmosphere for 1 d. Subsequently, DMF was evaporated, and the product was dialyzed against methanol for 1 d. Next, 100 mg NaN_3_ was dissolved in DMF and added to the mixture, and then the solution was stirred at 80 °C for 1 d. The product was purified by dialysis in methanol for 1 d before being dissolved in a mixture solution of THF and water (2:1). Then 200 mg PPh_3_ was added to the solution before the reaction was conducted at 45 °C for 2 d to reduce azido to amino groups. Finally, the solvent was evaporated, and the product was dialyzed against methanol for 1 d to obtain MLPG_A_, TLPG_A_, or WLPG_A_.

### Synthesis of TDPG_A_

DPG (Mw≈5,000 g mol^−1^) was synthesized by ring‐opening polymerization and DPG_A_ was synthesized through azidation and reduction reaction according to the literature.^[^ ^46^ ^]^ TDPG_A_ was synthesized via an amidation reaction between DPG_A_ polymers and T_LA_ nanosheets. 10 mg T_LA_ was dispersed in EDC (80 mg) solution in MES (0.2 M), and the solution was then added dropwise to 1 g DPG_A_ in MES (0.2 M). The reaction mixture was stirred at room temperature for 2 d before dialysis in Mili‐Q water (MWCO = 12–14 K) for 2 d to obtain TDPG_A_.

### Establishment of Mouse Model

Animal experiments were approved by the ethics committee of the Sixth Affiliated Hospital of Sun Yat‐sen University (No. IACUC‐2023021601). Adult female BALB/c mice (4 to 6 weeks, weight 18 to 22 g) were provided by YaoKang company (Guangdong). All mice were raised under specific pathogen‐free (SPF) facilities at Sixth Affiliated Hospital, Sun Yat‐sen University.

Model mice with eosinophilic airway inflammation were established according to the previous report.^[^ ^41^, ^42^ ^]^ BALB/c mice were intraperitoneally sensitized by 40 µg OVA in 100 µL PBS emulsified with 100 µL of alum adjuvant three times at 0th d, 7th d, and 14th d. Subsequently, the experimental mice were intranasally challenged with 500 µg OVA in 20 µL saline four times at 21st d, 22nd d, 23rd d, and 24th d. BALB/c mice sensitized and challenged with saline (same volume) were applied as sham mice.

### DNase I Treatment for Model Mice

A mouse model with eosinophilic airway inflammation and sham mice were established according to the above protocol. After that, DNase I (20 U) in saline (20 µL) was intranasally treated in the model mice at 4 h after every challenge. The sham mice and mice with eosinophilic inflammation treated with saline (20 µL) after every challenge were applied as negative control and positive control, respectively. 24 h after the last treatment, nasal lavage fluid (NALF) was collected from the experimental mice and cfDNA concentration was determined by pico‐green assay.

The nasal mucosa of the experimental mice was collected, and the total mRNA of the nasal mucosa was extracted using TRIzol. Then the mRNAs were converted to cDNA with an iScript cDNA synthesis kit. Subsequently, the iTaq Universal SYBR Green Supermix was used to conduct qRT‐PCR, and Ct values for cytokines (IL‐4, IL‐5, and IL‐6) were normalized to GAPDH. All reactions used standard cycling conditions with a melting temperature of 60 °C. The primers for each cytokine were designed based on the PrimerBank database and purchased from IDT (Table S1, Supporting Information).

After that, the mice were sacrificed by decapitation after the last treatment, the heads were quickly dissected to isolate the anterior part of the snout, including the nasal cavities. Subsequently, the maxillary bones (MBs) were fixed by immersion in 10% paraformaldehyde for 24 h followed by bone tissue decalcification in 10% EDTA for 3 weeks. Finally, the specimens were carefully rinsed in water, dehydrated in dry ethanol, and embedded in paraffin. The sections were cut, dewaxed, and stained with ECP.

### Biodistribution of LPG_A_ and TLPG_A_

Sham mice and model mice with eosinophilic inflammation were intranasally instilled with saline, LPG_A_‐Cy5 (100 µg), or TLPG_A_‐Cy5 (100 µg), respectively. The MBs, as well as major organs (hearts, lungs, livers, spleens, and kidneys) of the experimental mice, were excised and imaged at 1 d, 3 d, 5 d, and 7 d after treatment to study the biodistribution of polymers and nanosheets. In addition, the serum of mice was collected at the same time and the fluorescence intensity was determined to calculate the content of polymers and nanosheets in blood.

### Mouse Model Treatment with LPG_A_ and TLPG_A_

Sham mice and the model mice with eosinophilic inflammation were established. LPG_A_ (100 µg) or TLPG_A_ (100 µg) in 20 µL of saline was instilled at 4 h after every challenge. The sham mice and inflammatory model mice instilled with 20 µL of saline were considered as NC and PC, respectively. 24 h after the last treatment, NALF of the experimental mice was collected and cfDNA concentration was determined by pico‐green assay. The levels of cytokines (IL‐4, IL‐5, and IL‐6) in NALF were measured with corresponding ELISA kits. In another test, the nasal mucosa of the experimental mice was collected, and the total mRNA was extracted and converted to cDNA. Subsequently, qRT‐PCR was performed to determine the Ct values for cytokines (IL‐4, IL‐5, IL‐6, IL‐17, TNF‐α, and IFN‐γ), and the values were normalized to GAPDH. The gene sequences of each cytokine in the qRT‐PCR experiments were listed in Table S1 (Supporting Information).

### Nasal Mucosa Staining Analysis

The experimental mice were sacrificed and decapitated 24 h after treatment, The head was quickly dissected to isolate the anterior part of the snout and the nasal cavities. Subsequently, the bone tissues in MBs were decalcified for 3 weeks in EDTA solution after fixation for 24 h. After that, the specimens were carefully rinsed in water followed by dehydrated in dry ethanol and embedded in paraffin. Finally, the sections were cut, dewaxed, and treated with H&E and PAS staining. In addition, eosinophil staining and ECP immunofluorescence staining were also applied to these sections.

### Lung Inflammation Analysis

The experimental mice were sacrificed and decapitated at 24 h after treatment. After execution, the BALF was collected with an integrated catheter, and inflammatory cytokines (IL‐4, IL‐5, and IL‐6) levels were determined by ELISA. In addition, the lungs of the experimental mice were collected and fixed in 4% paraformaldehyde for 24 h. After that, H&E staining, ECP immunostaining, and PAS staining were applied to analyze the EET generation and inflammatory status in the lungs.

### Biosafety Evaluation of LPG_A_ and TLPG_A_

The biosafety of LPG_A_ and TLPG_A_ was evaluated with healthy mice. 300 µg LPG_A_ and TLPG_A_ were i.n. administered to the healthy mice once per day and four times in total. Half of the mice were sacrificed on the 14th d and the other half was sacrificed on the 28th d after the last treatment. Major organs (hearts, livers, lungs, spleens, and kidneys) and MBs were harvested and fixed in 4% paraformaldehyde for 24 h. Subsequently, the MBs were decalcified in 10% EDTA for 3 weeks and then embedded in paraffin. Finally, the tissue slices were prepared and stained with H&E for biosafety evaluation. In addition, PAS staining and ECP immunofluorescence staining were also applied to examine the inflammation in the nasal mucosa.

### Statistical Analysis

Data were displayed as mean ± S.D. Differences between groups were analyzed using Student's *t*‐test or one‐way analysis of variance when comparing two groups or more than two groups. GraphPad Prism 8 was used to perform the statistical analysis. In the figures, asterisks represent the following p values: *p<0.05, **p<0.01, and ***p<0.001.

## Conflict of Interest

The authors declare no conflict of interest.

## Supporting information

Supporting Information

## Data Availability

The data that support the findings of this study are available from the corresponding author upon reasonable request.
