# Supplementary material for: Functional 2D Nanoplatforms Alleviate Eosinophilic Chronic Rhinosinusitis by Modulating Eosinophil Extracellular Trap Formation
Source: Adv Sci (Weinh). 2024 Mar 13;11(19):2307800. doi: 10.1002/advs.202307800 (PMC11109617; doi:10.1002/advs.202307800)
Supplement: Supplementary file 1 — Supporting Information [file ADVS-11-2307800-s001.pdf]

ADVANCED SCIENCE
Open Access

Supporting Information

for *Adv. Sci.*, DOI 10.1002/advs.202307800

Functional 2D Nanoplatforms Alleviate Eosinophilic Chronic Rhinosinusitis by Modulating Eosinophil Extracellular Trap Formation

Zhaoxu Tu, Ming Liu, Changyi Xu, Yi Wei, Tong Lu, Yongqiang Xiao, Hongxia Li, Shuaiyin Zhang, Xinran Xie, Jian Li* and Weiping Wen**

Supporting Information

Functional 2D Nanoplatforms Alleviate Eosinophilic Chronic Rhinosinusitis by Modulating Eosinophil Extracellular Trap Formation

Zhaoxu Tu, Ming Liu, Changyi Xu, Yi Wei, Tong Lu, Yongqiang Xiao, Hongxia Li, Shuaiyin Zhang, Xinran Xie, Jian Li*, and Weiping Wen**

Z. Tu, M. Liu, H. Li, S. Zhang, X. Xie, W. Wen
Department of Otolaryngology, The Sixth Affiliated Hospital, Sun Yat-sen University, Guangzhou 510655, Guangdong, China

Y. Wei, T. Lu, J. Li and W. Wen
Department of Otolaryngology, The First Affiliated Hospital, Sun Yat-sen University, Guangzhou 510655, Guangdong, China

Z. Tu, M. Liu, C. Xu, H. Li, S. Zhang, X. Xie, W. Wen
Biomedical Innovation Center, The Sixth Affiliated Hospital, Sun Yat-sen University, Guangzhou 510655, Guangdong, China

C. Xu
Department of Clinical Laboratory, The Sixth Affiliated Hospital, Sun Yat-sen University, Guangzhou 510655, Guangdong, China

Y. Xiao
ENT Institute, Eye & ENT Hospital, Fudan University, Shanghai 201114, China

Table of contents

1. Experimental ... 3
2. The synthesis routes of $MLPG_A$, $TLPG_A$, and $WLPG_A$ ... 9
3. The synthesis routes of $TDPG_A$ ... 10
4. NMR characterizations of $TLPG_A$ ... 11
5. FTIR characterizations of $TLPG_A$ ... 12
6. UV-vis characterizations ... 13
7. TEM characterizations of $TDPG_A$ ... 15
8. Cytotoxicity tests ... 16
9. DLS and zeta potential ... 17
10. cfDNA binding studies ... 18
11. Protein adsorption tests ... 19
12. Comparison of TLR9 activation and EET formation ... 20
13. Quantitative biodistribution studies ... 21
14. Analysis of the cytokines level in nasal mucosa by qRT-PCR ... 22
15. Comparison of in vivo anti-inflammation effect ... 23
16. Analysis of the cytokines level in NALF by ELISA ... 24
17. Analysis of the IL-4 level in nasal mucosa by immunostaining ... 25
18. Analysis of the eosinophil number in nasal mucosa ... 26
19. Analysis of the inflammation in lungs ... 27
20. In vivo biocompatibility tests ... 29
21. References ... 31

1. Experimental

Materials: 1,1,1-tris(hydroxymethyl)propane, potassium methylate, glycidol, triethylamine (TEA), sodium azide (NaN_3), methanesulfonyl chloride (MsCl), triphenylphosphine (PPh_3), ethyl vinyl ether (EVE), P-toluenesulfonic acid (PTSA), calcium hydride (CaH_2), tetrabutylammonium azide (Nbu_4N_3), triisobutylaluminum (TIBAL), were purchased from Sigma-Aldrich and used directly without any further purification. Molybdenum sulfide (MoS_2) powder, titanium sulfide (TiS_2) powder, tungsten sulfide (WS_2) powder, n-butyllithium solution (2.0 M in cyclohexane), lipoic acid (LA), 1-ethyl-3-(3-dimethyllaminopropyl)carbodiimide hydrochloride (EDC.HCl), *N-hydroxysuccinimide* (NHS), methanol, N,N-dimethylformamide (DMF) and tetrahydrofuran (THF), N-Methyl pyrrolidone (NMP), *dimethyl* sulfoxide (DMSO) were purchased from Sigma-Aldrich. Quant-iT PicoGreen DNA assay kit, QUANTI-Blue™ for alkaline phosphatase detection, 4',6-diamidino-2-phenylindole (DAPI), n-hexane, and TRIzol reagent were purchased from Fisher Scientific. Dermatophagoides pteronyssinus allergen 1 (Derp1) was bought from INDOOR Biotechnologies. Bovine serum albumin (BSA), ODN1826, ODN2088, lipopolysaccharide (LPS), ovalbumin (OVA), alum adjuvant, polyamidoamine generation 3.0 (P-G3), and Cell Counting Kit-8 assay (Dojindo Molecular Technologies, Inc., Rockville, MD, USA) were purchased from Millipore-Sigma (US). Human and murine IL-4, IL-5, and IL-6 ELISA kits were purchased from Invitrogen (US). Fluorescein Isothiocyanate isomer I (FITC) and Cyanine 5 (Cy5)-NHS ester were purchased from Lumiprobe Corporation (FL, USA). iScript cDNA synthesis kit and iTaq Universal SYBR Green Supermix were bought from Bio-Rad. Milli-Q water was applied in all experiments.

Equipment: Nuclear magnetic resonance spectroscopy (NMR) spectra were measured with a Jeol Eclipse (USA) nuclear magnetic resonance spectrometer (500 MHz). Ultraviolet-visible spectrophotometry (UV-vis) absorption spectra were measured with a U-3310 spectrophotometer (Hitachi, Japan). Zeta potential and dynamic light scattering (DLS) data

were recorded on a Malvern NANO ZSPO in corresponding conditions. Fourier transform infrared spectroscopy (FTIR) spectra were recorded on a Jasco FT/IR-4100 spectrometer. Transmission electron microscopy (TEM) was performed with a FEI Tecnai G2 F30 TEM. Fluorescence was measured by a JASCOFP-6500 Spectrofluorometer. Quanti-Blue and CCK8 assays were performed using a microplate reader (Bio-Tek, Winooski, VT). Flow cytometer was performed using a BD FACS caliber flow cytometer. Quantitative polymerase chain reaction (qPCR) was measured with LightCycler 480 II (Roche, US). Confocal laser scanning microscopy (CLSM) experiments were conducted with a Nikon Ti Eclipse inverted microscope with an A1 scanning confocal unit and quantification of the specified color area was calculated by Image J software. H&E, PAS staining slices were scanned using an automated slice scanning system (AxioScan.Z1, Zeiss). Biodistribution fluorescent images were recorded with an IVIS Spectrum system (PerkinElmer, USA).

Biodegradation tests: 3 mg MoS_2, TiS_2, WS_2, $MLPG_A$, $TLPG_A$, $WLPG_A$, and $TDPG_A$ were dispersed in 3 mL PBS (7.4) and then evenly divided into three dialysis tubes (MWCO=2k), respectively. After that, the above solutions were subjected to shaking at 37ºC for 4 weeks, and the UV absorbance (360 nm) was recorded at the beginning, 1 week, 2 weeks, 3 weeks, and 4 weeks after incubation. The degradation of nanosheets was calculated by comparing the absorbance value (360 nm) of the solutions with the initial absorbance value (360 nm) before the experiments. Additionally, the nanoscale morphologies of nanosheets before and after degradation were also recorded by TEM.

cfDNA binding assay: cfDNA concentration was determined by pico-green assay, a fluorescent dye that could specifically bind cfDNA.[1,2] The cfDNA binding efficacy of LPG_A, DPG_A, $MLPG_A$, $TLPG_A$, $WLPG_A$, $TDPG_A$ and P-G3 were determined by pico-green competitive binding tests. Firstly, diluted pico-green reagent and cfDNA from calf thymus were mixed in

Mili-Q water or FBS (10%) aqueous solution and incubated for 30 min at 37ºC in a dark environment. After that, LPG_A, DPG_A, $MLPG_A$, $TLPG_A$, $WLPG_A$, $TDPG_A$ and P-G3 were added to the mixture with nanomaterials/cfDNA mass ratios from 0.0625 to 32 before the mixture was shaken and incubated for 30 min at 37ºC in dark environment. Finally, the fluorescence intensity was measured with a Multiwall Plate Reader (Excitation: 480 nm; Emission: 520 nm). The binding efficiency of LPG_A, DPG_A, $MLPG_A$, $TLPG_A$, $WLPG_A$, $TDPG_A$ and P-G3 to cfDNA was calculated by comparing the fluorescent intensity after incubation to the initial value.

Protein adsorption determination: The protein adsorption determination was performed according to a reported method.[3] LPG_A, DPG_A, $MLPG_A$, $TLPG_A$, $WLPG_A$, $TDPG_A$, and P-G3 solutions (1 mL, 25 to 100 µg/mL) were mixed with BSA-FITC (1 mL, 100 µg/mL). The mixed solution was then centrifugated at 11,000 rpm for 10 min after stirring at 37 °C for 30 min. The supernatant was carefully collected, and the fluorescent intensity was measured to calculate the protein concentration. The protein adsorption (PA) was calculated using the following equation: $PA = (C1 - C2)/C1 \times 100\%$. In this equation, C1 and C2 are the initial concentration of BSA and the BSA concentration in the supernatant after centrifugation, respectively.

Cytotoxicity test: BEAS-2B cells were cultured in Dulbecco's modified Eagle's medium (DMEM) with 10% Fetal Bovine Serum (FBS) and 1% Penicillin-Streptomycin (PS) at 37°C in a humidified atmosphere with 5% CO_2. The cytotoxicities of the LPG_A, DPG_A, $MLPG_A$, $TLPG_A$, $WLPG_A$ and $TDPG_A$ against BEAS-2B cells were evaluated with CCK-8 assay. Firstly, BEAS-2B cells (5×10^3/well) were seeded in a 96-well plate and cultured until the cell density reached 70–80%. After that, the culture medium was replaced by fresh medium with different concentrations of LPG_A, DPG_A, $MLPG_A$, $TLPG_A$, $WLPG_A$ and $TDPG_A$. Subsequently, the medium was replaced again with fresh medium containing 10% CCK-8 reagent after 24 h or

48 h incubation. Finally, the plates were incubated for another 2-3 h at 37°C and then the absorbance (450 nm) of the wells was recorded using a Multiplate Reader. The cytotoxicity of LPG_A, DPG_A, $MLPG_A$, $TLPG_A$, $WLPG_A$ and $TDPG_A$ was calculated by comparing the absorbance value to the value of cells treated with medium-only.

TLR9 activation with QUANTI-Blue assay: HEK-blue™ hTLR9 (HEK-TLR9) reporter cell lines were obtained from InvivoGen, San Diego, CA, and were cultured following the manufacturer's protocol. The HEK-TLR9 cells were originated and constructed by co-transfection of HEK 293 cells with the hTLR9 gene and optimized embryo-secreted alkaline phosphatase (SEAP) reporter gene.[4] The SEAP activity was determined using the alkaline phosphatase (ALP) detection reagent, QUANTI-Blue™ and calculated by optical density (OD) at 620 nm. ODN1826 as non-methylated DNA and synthetic analogs of cfDNA, was applied as TLR9 agonists. HEK-TLR9 cells were seeded into 96-well plates (8×10^4/well) and the cells were attached to the wall after 6–8 h incubation. After that, ODN1826, ODN1826+LPG_A, ODN1826+DPG_A, ODN1826+$MLPG_A$, ODN1826+$TLPG_A$, ODN1826+$WLPG_A$ and ODN1826+$TDPG_A$ were added to the wells and incubated for the following 24 h. ODN1826 was set as 1 mg/mL and the concentration of nanomaterials was from 0.5 to 4 μg/mL. The cells incubated with medium-only or ODN1826-only were considered as negative control (NC) and positive control (PC), respectively. Finally, 50 μL supernatants of each well were harvested and mixed with 150 μL QUANTI-Blue™ medium. The new plate was incubated for 2 h and TLR9 activation was determined by the absorbance (620 nm) value measured using a Multiwall Plate Reader.

Inhibition of the nasal secretion-induced TLR9 activation: HEK-TLR9 cells were incubated with nasal secretions from ECRS patients in a 96-well plate, and the concentration of nasal secretions was set as 1 μg/mL (cfDNA concentration). LPG_A, $MLPG_A$, $TLPG_A$ and $WLPG_A$

were added to the wells at 2 μg/mL and 4 μg/mL, respectively and the cells were incubated for 24 h. The cells incubated with medium-only and nasal secretion-only were considered as NC and PC. respectively. Then 50 μL culture supernatants were collected and added to 150 μL QUANTI-Blue™ solution in a new plate. After incubation for another 2 h, TLR9 activation was determined by the absorbance (620 nm) value measured using a Multiwall Plate Reader.

Derp1 and LPS-induced cfDNA release: BEAS-2B cells (5×10^3/well) were seeded in a 96-well plate and incubated at 37°C until the cell density reached 70-80%. In the next step, Derp1 and LPS (1 μg/mL) were added into the medium and the cells were incubated for another 12 h. Subsequently, the culture medium was replaced by fresh medium with LPG_A or $TLPG_A$ (2 μg/mL or 4 μg/mL) and the cells were cultured for another 12 h. The cells incubated with medium-only during the whole process were considered as NC. The cells incubated with Derp1 or LPS in the first stage and medium-only in the second stage were considered as PC. Finally, the conditioned medium was collected and cfDNA concentrations were measured with a pico-green assay.

TLR9 activation by conditioned medium: HEK-TLR9 cells were incubated with Derp1 or LPS-conditioned medium and cfDNA concentration is 1 μg/mL. Subsequently, LPG_A or $TLPG_A$ (2 μg/mL or 4 μg/mL) were added into the cells and incubated for another 24 h. The cells incubated with only fresh medium or conditioned medium were considered NC and PC, respectively. Finally, culture supernatants (50 μL) were harvested and added to QUANTI-Blue™ solution (150 μL) in a transparent plate and incubated for another 2 h. TLR9 activation was determined by the absorbance (620 nm) value measured using a Multiwall Plate Reader.

ODN1826-induced EET formation: Human peripheral blood eosinophils were isolated from ECRS patients with a MACSxpress® eosinophil isolation kit (Stem Cell, Canada).[5]

Eosinophils were cultured using Eosinophils Medium W/Kit in 24-well plates with coverslips on the bottom of wells. The eosinophils were split into two groups: one group of cells was cultured in medium-only and the other group was pretreated with ODN2088 for 1 h. After that, ODN1826 was added to these cells and incubated for another 4 h to induce EET formation. Subsequently, DAPI staining and eosinophil cationic protein (ECP) immunostaining were applied to identify the EETs on the coverslips. The EET formation was observed using confocal laser scanning microscopy (CLSM) and the EETs area in the images was qualitatively calculated by ImageJ software.

Conditioned medium-induced EET formation: Human eosinophils isolated from ECRS patients were cultured using Eosinophils Medium W/Kit in 24-well plates with coverslips on the bottom of wells. After that, Derp1 or LPS-conditioned medium was added to these cells and LPG_A or $TLPG_A$ (2 μg/mL or 4 μg/mL) was also added to examine their suppression efficacy for EET formation. After 4 h incubation, EETs on the coverslips were identified by DAPI staining and ECP immunostaining. The EET formation was observed by CLSM and qualified by ImageJ software.

Clinical samples: 20 nasal secretions were collected with an expansive sponge from the ECRS patients and 20 control nasal secretions were donated by healthy volunteers in the Sixth Affiliated Hospital of Sun Yat-sen University and the First Affiliated Hospital of Sun Yat-sen University. Nasal secretions collections were approved by the ethics committee of Sun Yat-sen University (2023ZSLYEC-497).

2. The synthesis routes of $MLPG_A$, $TLPG_A$, and $WLPG_A$

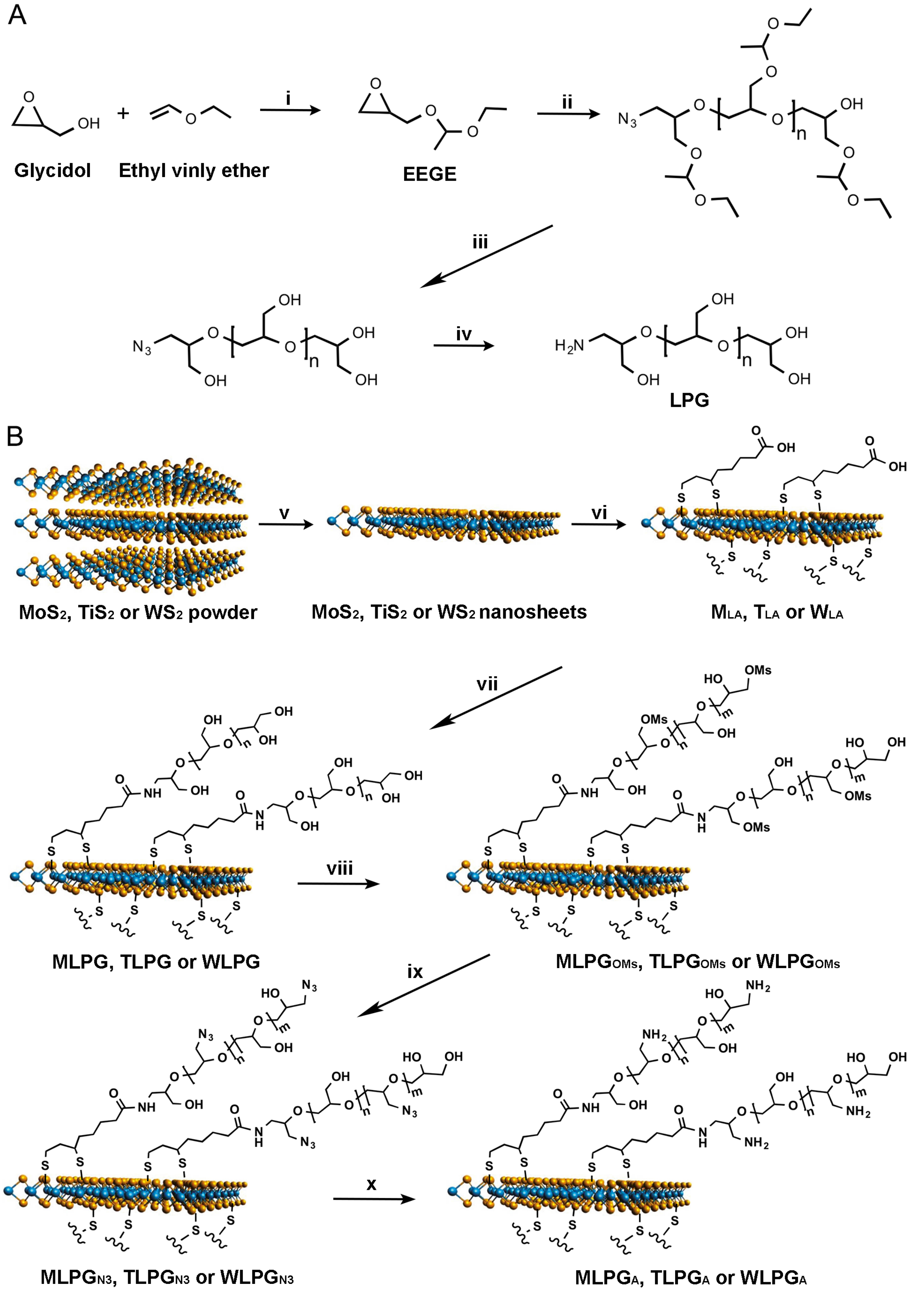

Figure S1. (A) Synthesis routes of LPG. (B) Synthesis routes of $MLPG_A$, $TLPG_A$, and $WLPG_A$.

Detailed protocols are provided in the Experimental section.

3. The synthesis routes of $TDPG_A$

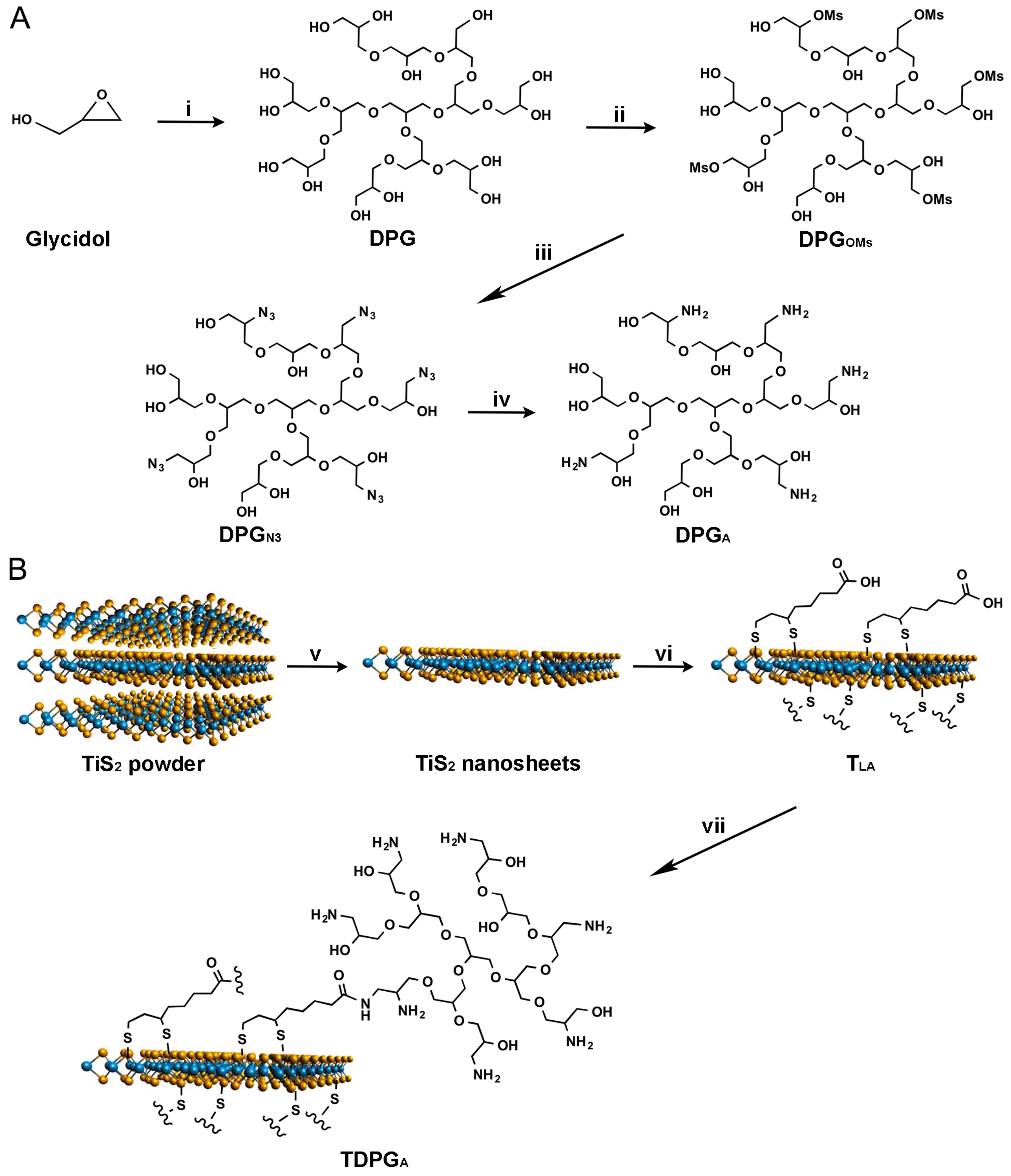

Figure S2. (A) Synthesis routes of DPG_A. (B) Synthesis routes of $TDPG_A$. Detailed protocols are provided in the Experimental section.

4. NMR characterizations of TLPG$_A$

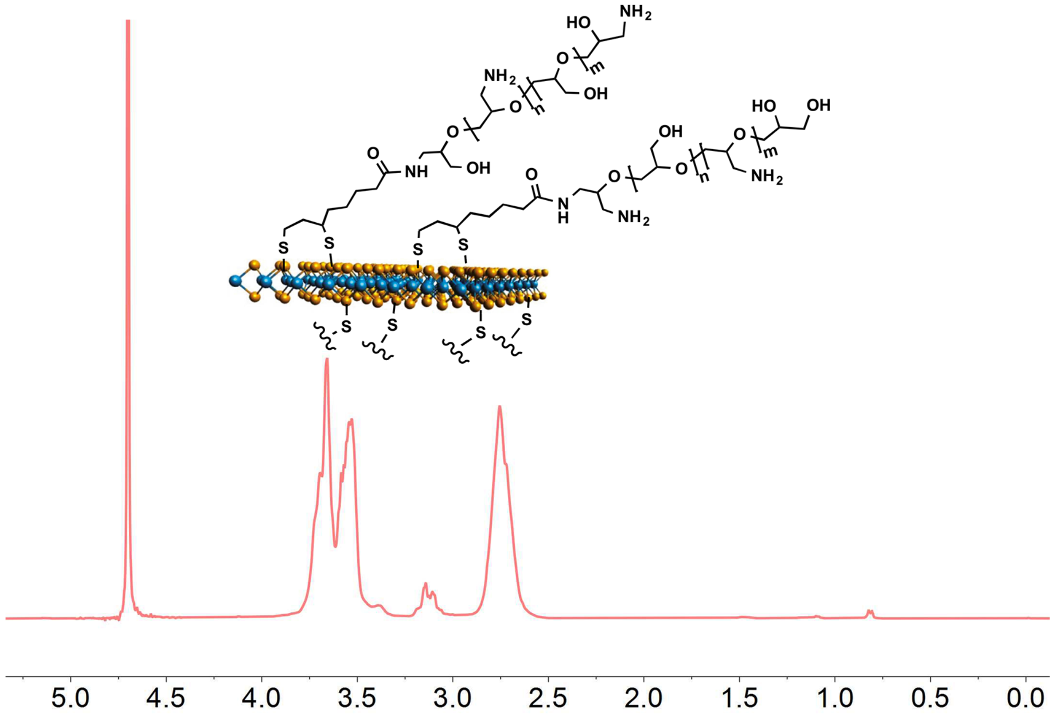

Figure S3. ^{1}H NMR data of TLPG$_A$ in D_2O. Signals at 4.7 ppm are attributed to the protons of D_2O.

5. FTIR characterizations of $TLPG_A$

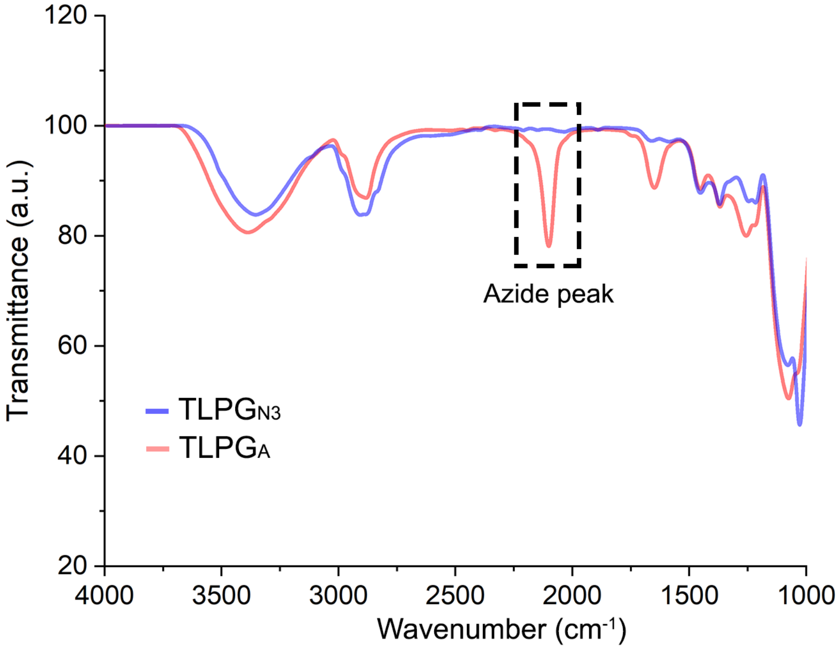

Figure S4. The FTIR spectra of $TLPG_{N3}$ and $TLPG_A$. The peak around 2100 cm^{-1} was attributed to the azide groups. The reduction of azide to amino groups was followed by the disappearance of the azide peak.

6. UV-vis characterizations

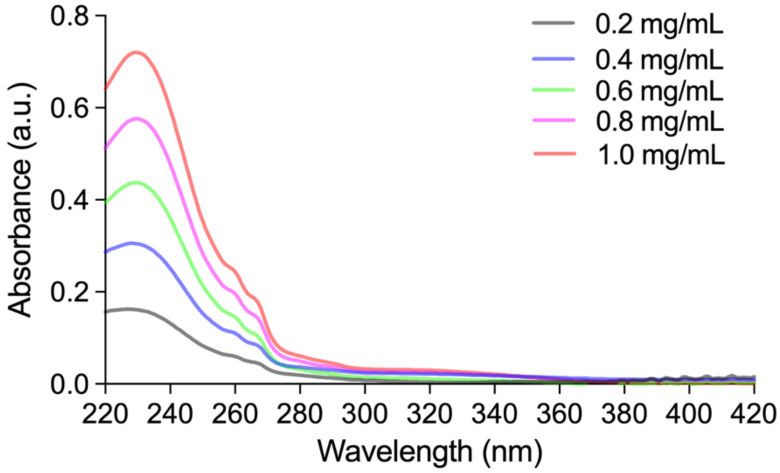

Figure S5. UV–vis absorbance of LPG_A at different concentrations from 0.2 to 1 mg/mL.

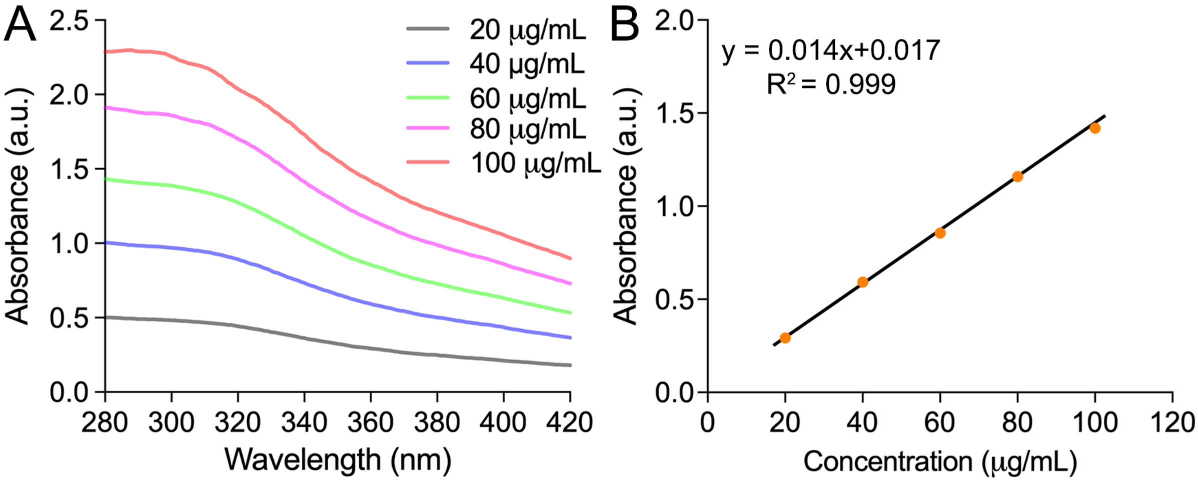

Figure S6. (A) UV–vis absorbance of MoS_2 at different concentrations from 20 to 100 µg/mL. (B) Standard curve of MoS_2 with absorbance at 360 nm.

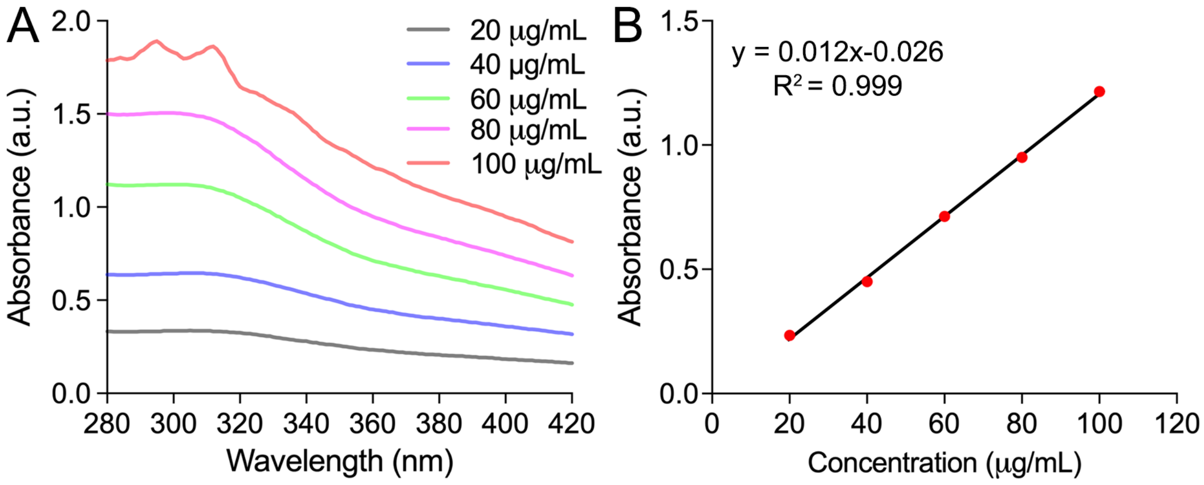

Figure S7. (A) UV–vis absorbance of TiS_2 at different concentrations from 20 to 100 μg/mL. (B) Standard curve of TiS_2 with absorbance at 360 nm.

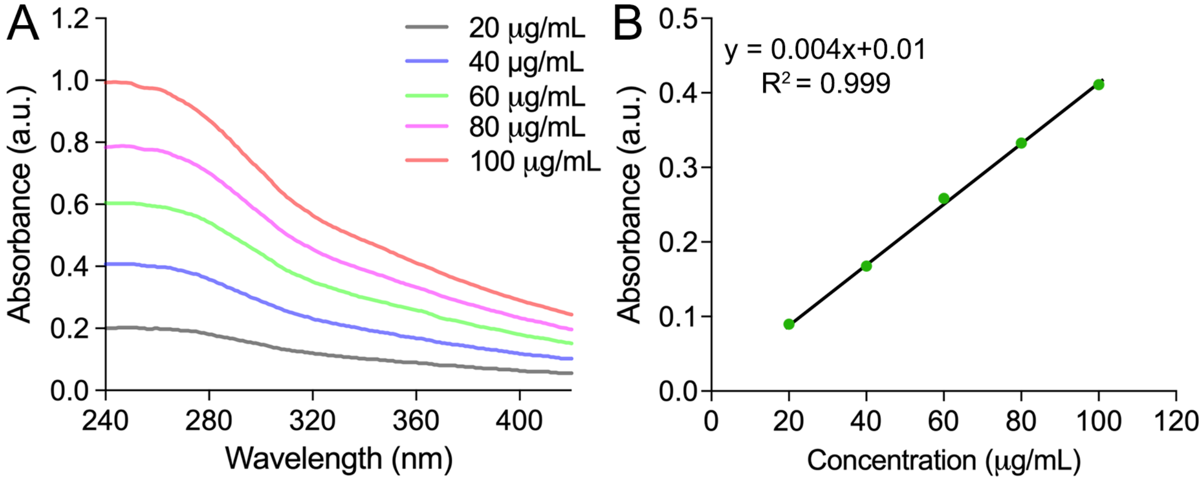

Figure S8. (A) UV–vis absorbance of WS_2 at different concentrations from 20 to 100 μg/mL. (B) Standard curve of WS_2 with absorbance at 360 nm.

7. TEM characterizations of $TDPG_A$

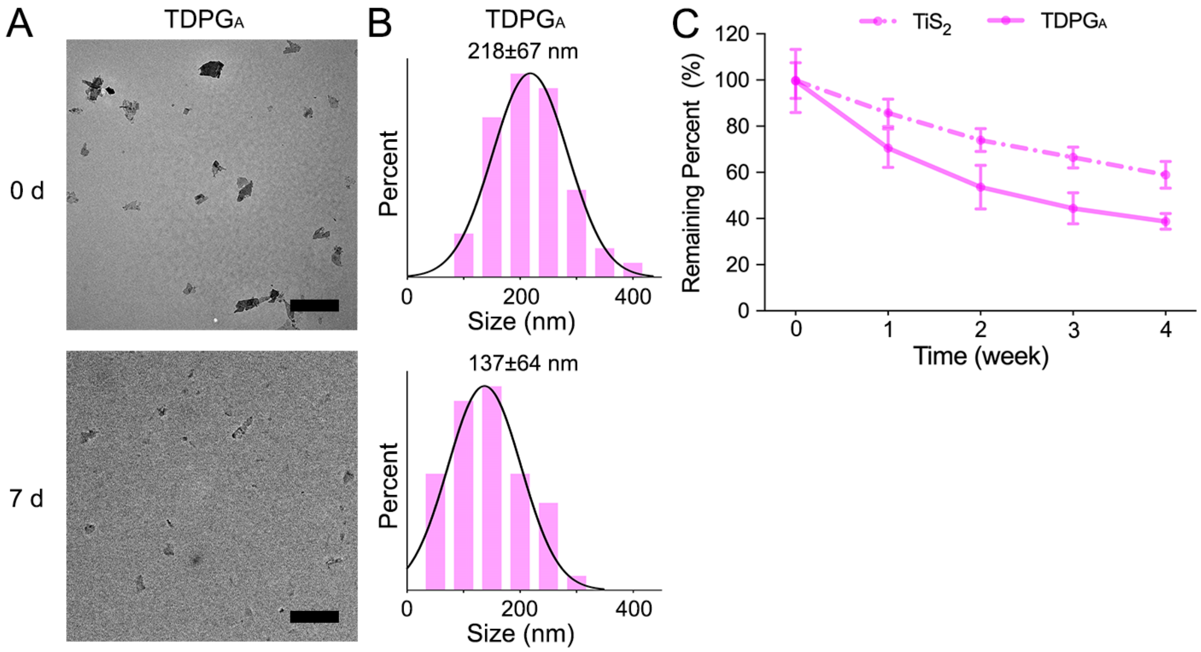

Figure S9. TEM characterizations of $TDPG_A$. (A) Representative TEM images of $TDPG_A$ before and after incubation in PBS (pH 7.4) at 37°C for 7 days. Scale bars: 500 nm. (B) Size profiles of $TDPG_A$ before and after incubation in PBS (pH 7.4) at 37°C for 7 days (based on the TEM images). (C) Relative absorbance (360 nm) of TiS_2 and $TDPG_A$ during a 4-week incubation in PBS (pH 7.4). Data represent the mean ± S.D. (n=3).

8. Cytotoxicity tests

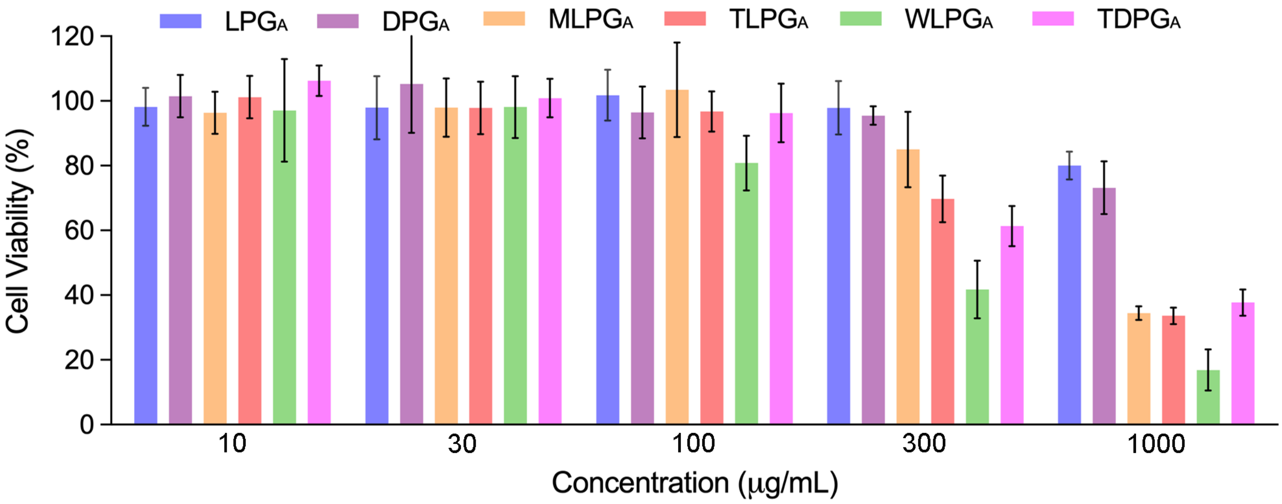

Figure S10. Viability of BEAS-2B cells treated for 48 hours with various concentrations of LPG_A, DPG_A, $MLPG_A$, $TLPG_A$, $WLPG_A$, and $TDPG_A$. Data represent mean ± S.D. (n=3, Student's t-test).

9. DLS and zeta potential

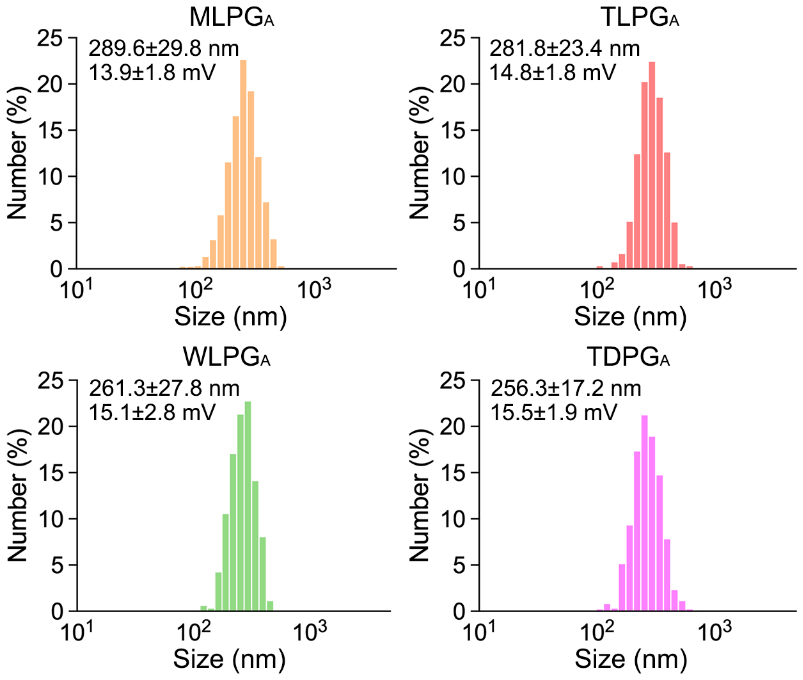

Figure S11. Dynamic light scattering (DLS) and zeta potential characterizations of MLPG$_A$, TLPG$_A$, WLPG$_A$, and TDPG$_A$ in PBS (7.4).

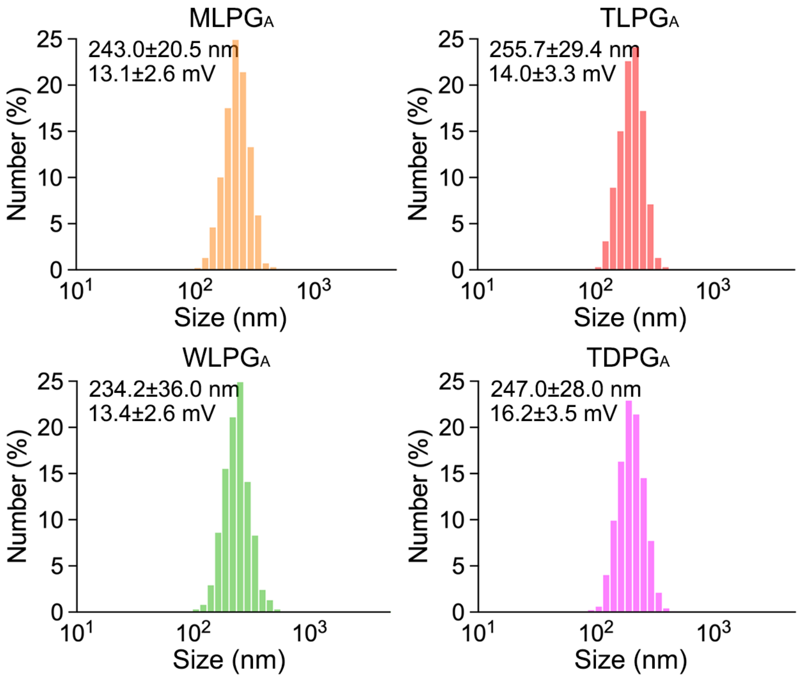

Figure S12. DLS and zeta potential characterizations of MLPG$_A$, TLPG$_A$, WLPG$_A$, and TDPG$_A$ after incubation in FBS (10%) for 3 d.

10. cfDNA binding studies

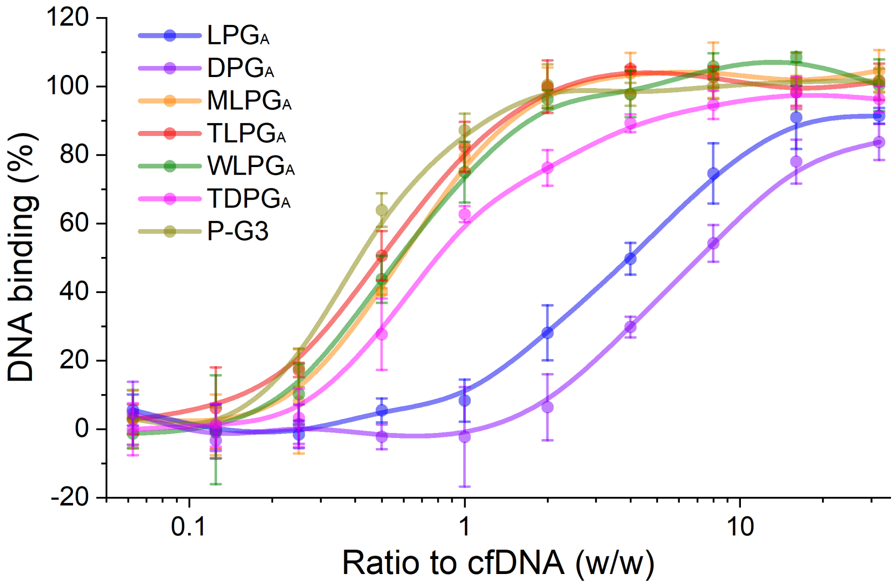

Figure S13. cfDNA binding efficiency of LPG$_A$, DPG$_A$, MLPG$_A$, TLPG$_A$, WLPG$_A$, TDPG$_A$, and P-G3 in water. Data represent mean ± S.D. (n=3, Student's t-test).

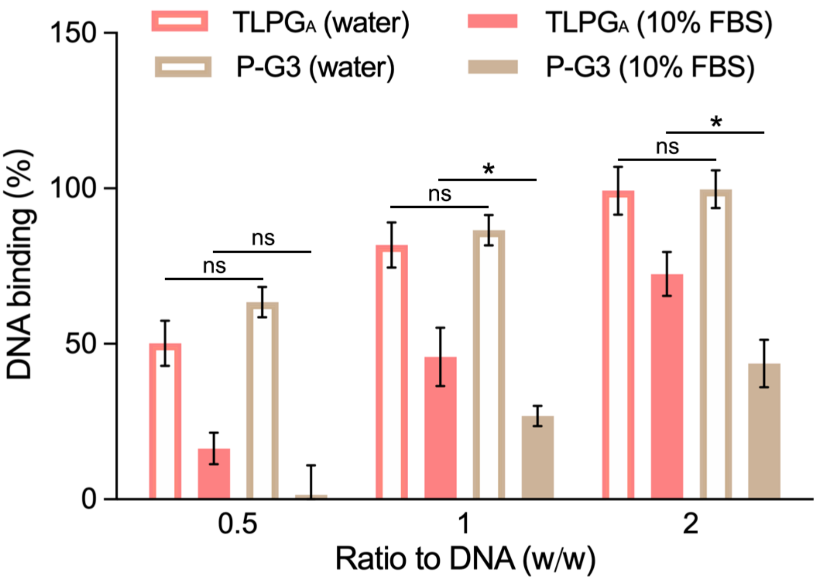

Figure S14. Comparison of cfDNA binding efficiency of TLPG$_A$ and P-G3 in water or FBS (10%) solutions. Data represent the mean ± S.D. (n=3, Student's t-test, ns: no significant difference, *P<0.05).

11. Protein adsorption tests

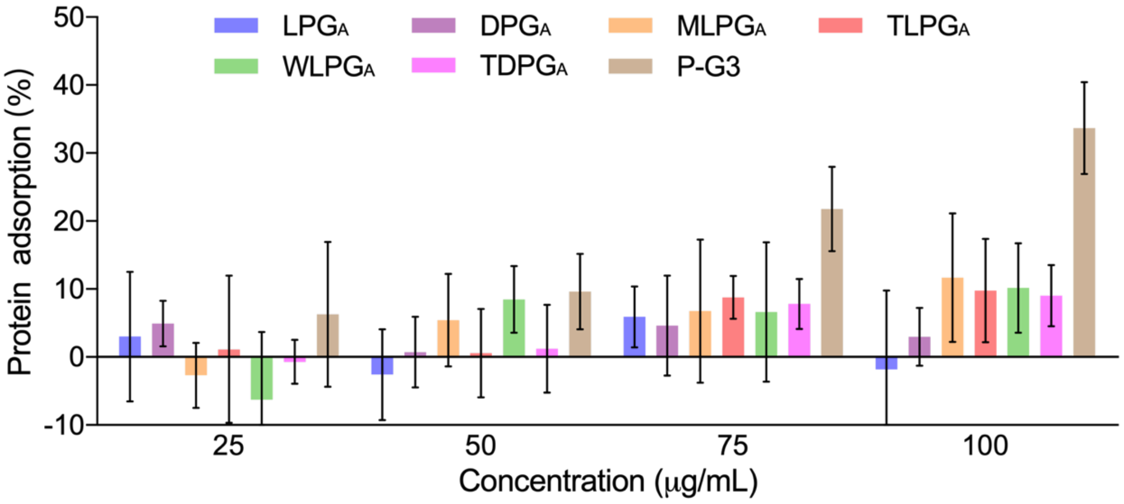

Figure S15. Protein adsorption of LPG_A, DPG_A, $MLPG_A$, $TLPG_A$, $WLPG_A$, $TDPG_A$, and P-G3 against bovine serum albumin (BSA). Data represent mean ± S.D. (n=3, Student's t-test).

12. Comparison of TLR9 activation and EET formation

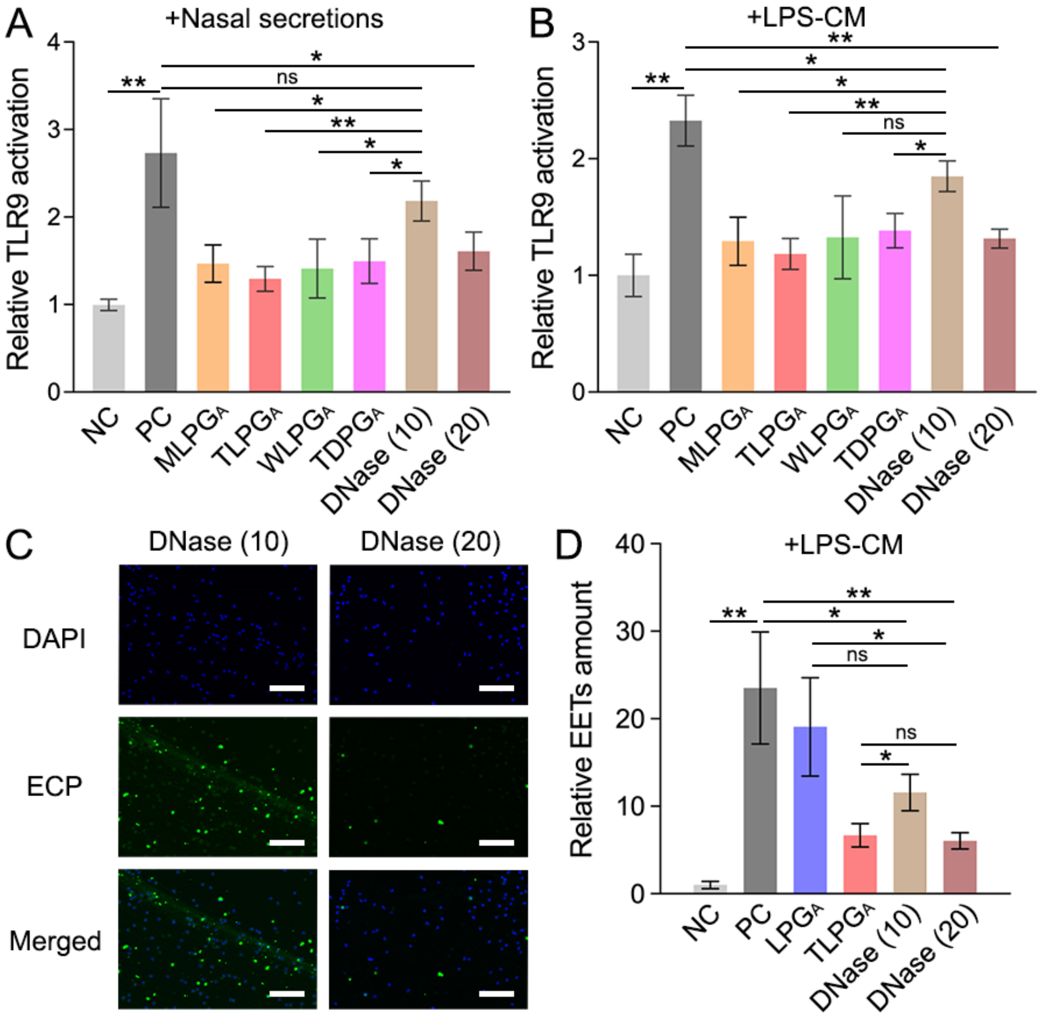

Figure S16. (A) Comparison of the nasal secretions (ECRS patients)-induced TLR9 activation of HEK-TLR9 cells after incubation with MLPG$_A$, TLPG$_A$, WLPG$_A$, TDPG$_A$, and DNase. (B) Comparison of the LPS-CM-induced TLR9 activation of HEK-TLR9 cells after incubation with MLPG$_A$, TLPG$_A$, WLPG$_A$, TDPG$_A$, and DNase. (C) Representative DAPI and ECP co-staining images of the LPS-CM-treated eosinophils after incubation with DNase. Scale bars: 100 µm. (D) Comparison of the EETs area in the representative CLSM images. Eosinophils incubated with medium-only or CM-only were considered as the NC or PC, respectively. Data represent the mean ± S.D. (n=3, Student's t-test, ns: no significant difference, *P<0.05, **P<0.01).

13. Quantitative biodistribution studies

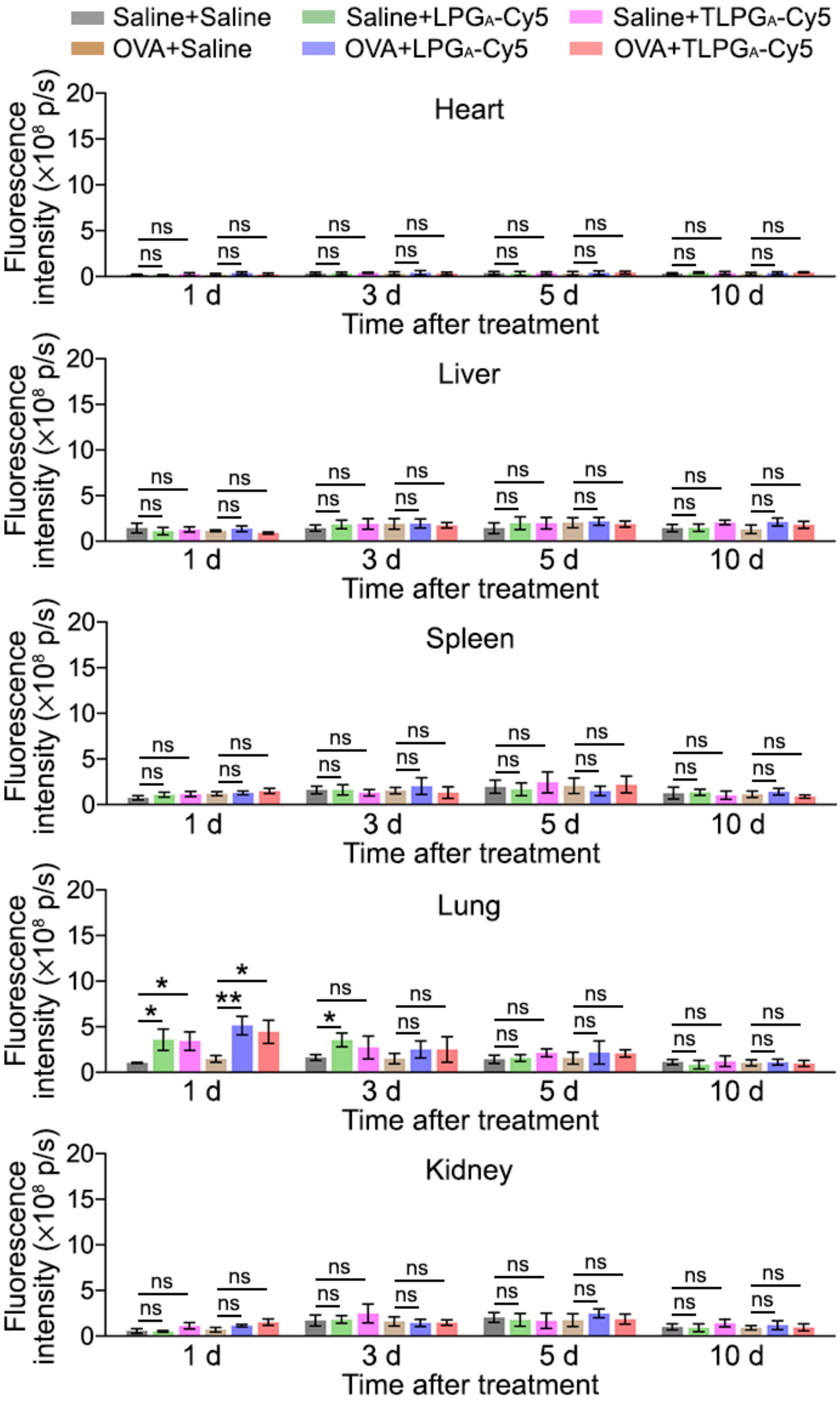

Figure S17. Quantification of Cy5 fluorescence intensity in hearts, livers, spleens, lungs and kidneys of experimental mice after intranasal instillation with LPG_A-Cy5 and $TLPG_A$-Cy5. Data indicate mean ± S.D. (ns represents no significant difference, $^*p < 0.05$, $^{**}p < 0.01$).

14. Analysis of the cytokines level in nasal mucosa by qRT-PCR

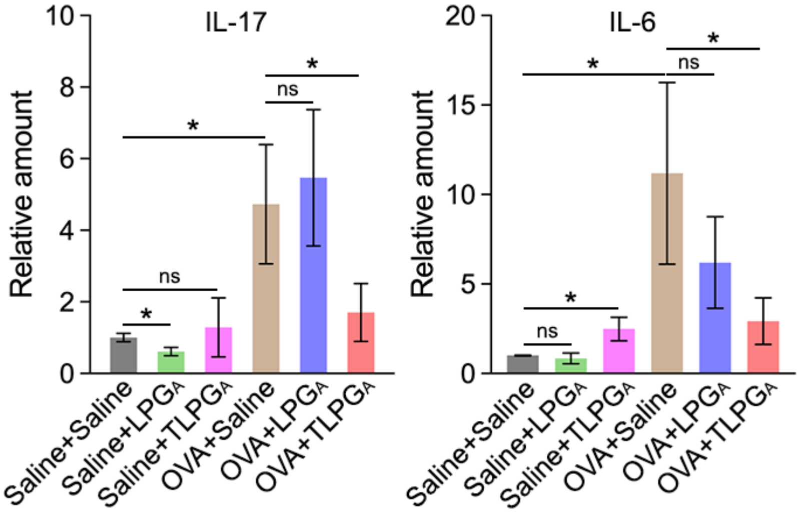

Figure S18. The relative mRNA expression level of IL-17, and IL-6 in the nasal mucosa of experimental mice. Data represent mean ± S.D. (ns represents no significant difference, $*p < 0.05$).

Table S1. Primer sequences in qRT-PCR experiments**.**

Species	Target	Forward primer (5' to 3')	Reverse primer (5' to 3')
Mice	GAPDH	AGGTCGGTGTGAACGGATTTG	TGTAGACCATGTAGTTGAGGTCA
Mice	IL-4	GGTCTCAACCCCCAGCTAGT	GCCGATGATCTCTCTCAAGTGAT
Mice	IL-5	TCAGGGGCTAGACATACTGAAG	CCAAGGAACTCTTGCAGGTAAT
Mice	IL-6	TAGTCCTTCCTACCCCAATTTCC	TTGGTCCTTAGCCACTCCTTC
Mice	IL-17	TATCCCTCTGTGATCTGGGAAG	ATCTTCTCGACCCTGAAAGTGA
Mice	TNF-α	CAGGCGGTGCCTATGTCTC	CGATCACCCCGAAGTTCAGTAG
Mice	IFN-γ	CTGGAGGAACTGGCAAAAGG	TGTGGGTTGTTGACCTCAAACT

15. Comparison of in vivo anti-inflammation effect

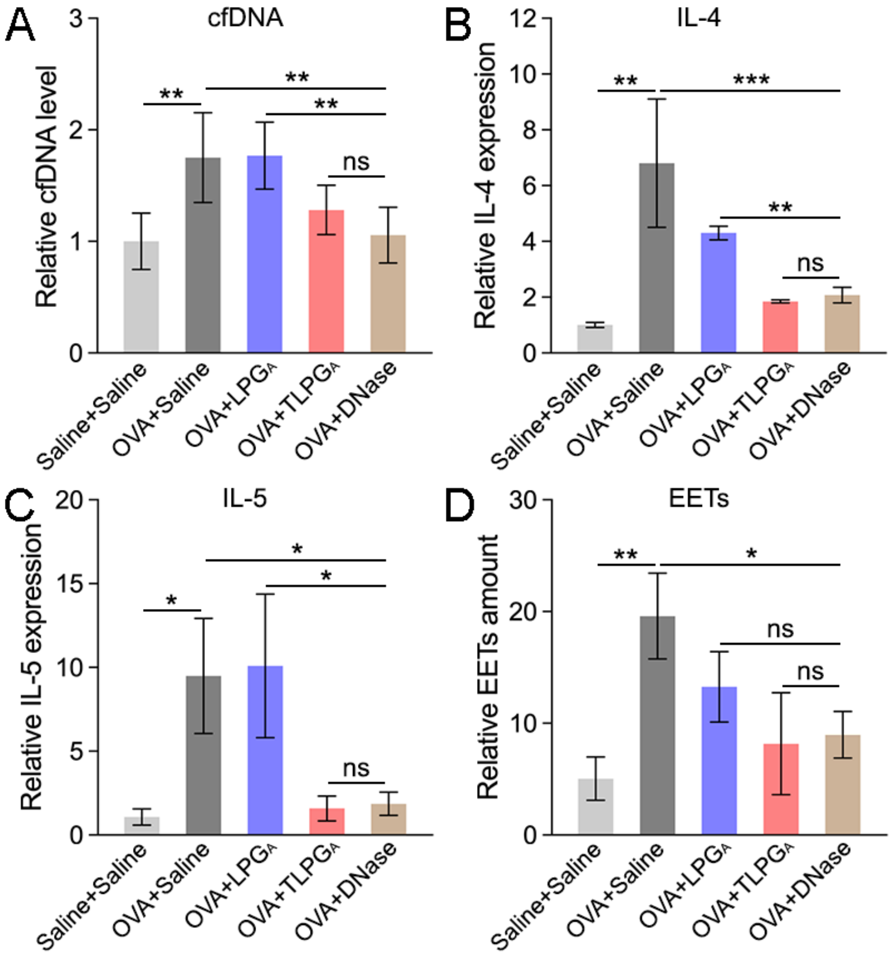

Figure S19. (A) Comparison of the cfDNA level in NALF of experimental mice. (B) Comparison of the IL-4 expression level in nasal mucosa of experimental mice. (C) Comparison of the IL-5 expression level in nasal mucosa of experimental mice. (D) Comparison of the EETs level in nasal mucosa of experimental mice. Data represent the mean ± S.D. (Student's t-test, ns represents no significant difference, *P<0.05, **P<0.01, ***P<0.001).

16. Analysis of the cytokines level in NALF by ELISA

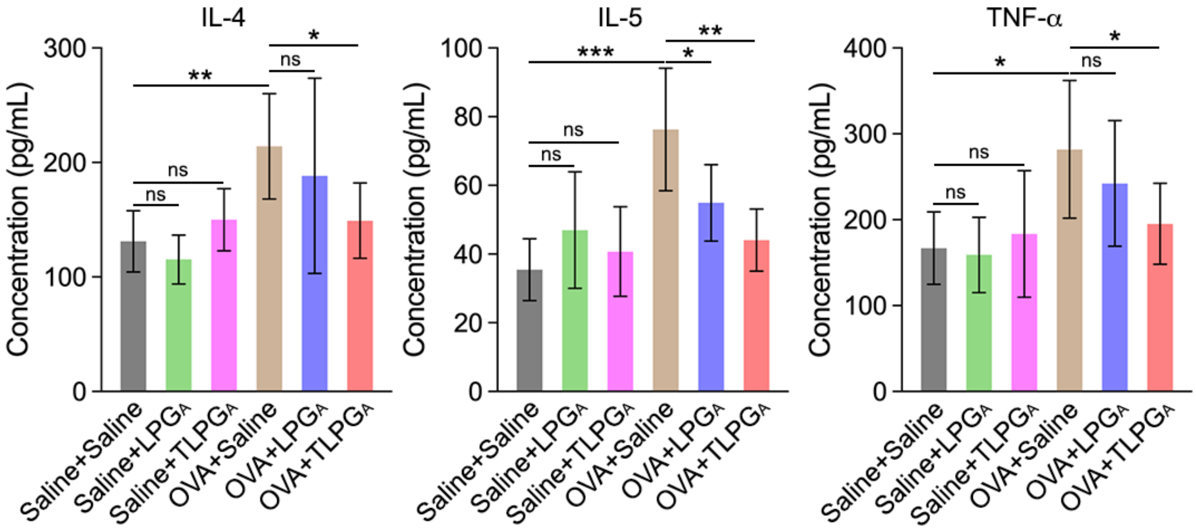

Figure S20. The IL-4, IL-5, and TNF-α concentration in NALF of mice following different treatments. Data represent mean ± S.D. (ns represents no significant difference, *p < 0.05, **p < 0.01, ***p < 0.001).

17. Analysis of the IL-4 level in nasal mucosa by immunostaining

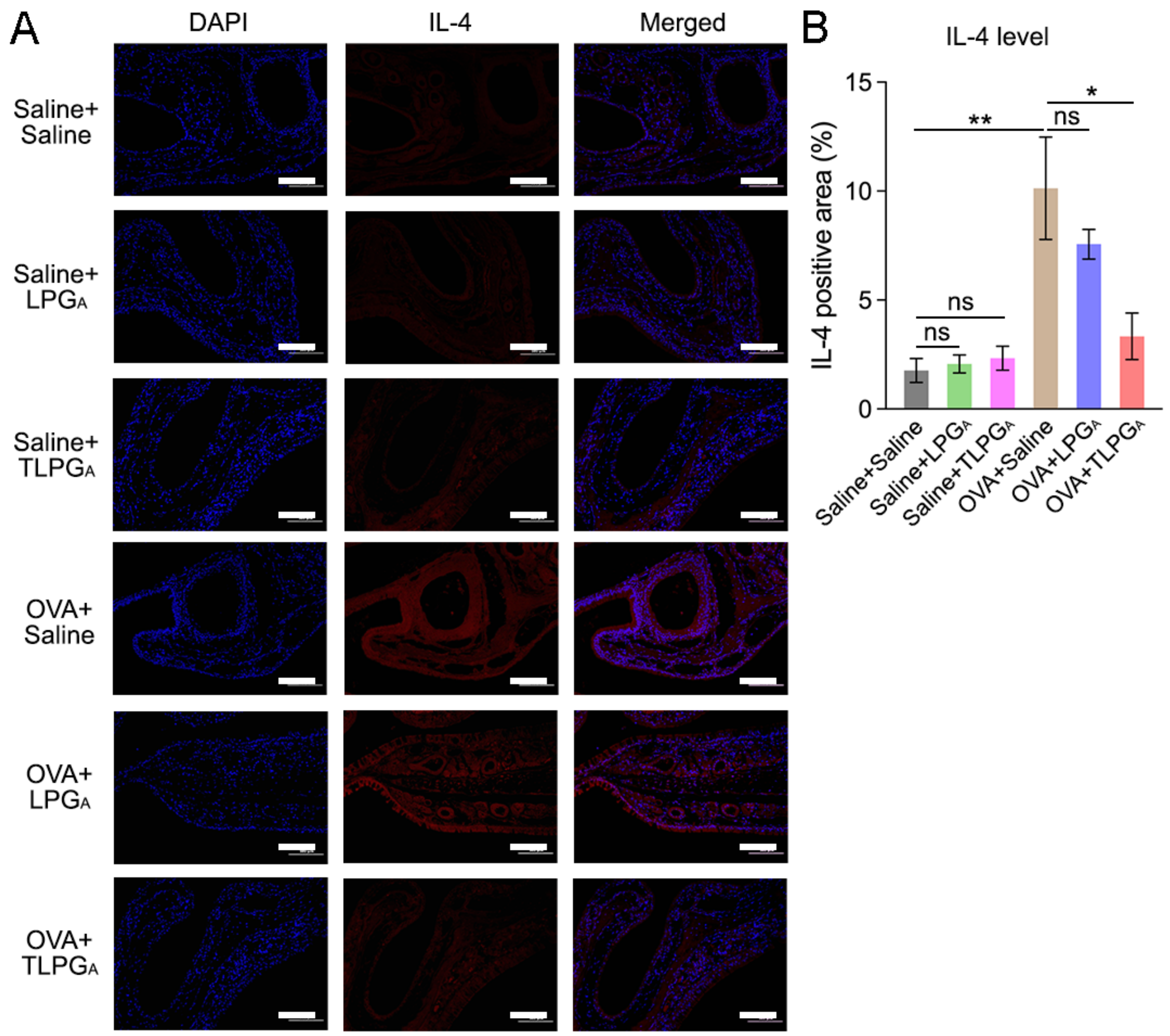

Figure S21. (A) Representative IL-4 antibody staining images of nasal mucosas from experimental mice. Scale bars: 100 μm. (B) Quantification of IL-4 positive area in the staining images. Data represent mean ± S.D. (ns represents no significant difference, *$p<0.05$, **$p<0.01$).

18. Analysis of the eosinophil number in nasal mucosa

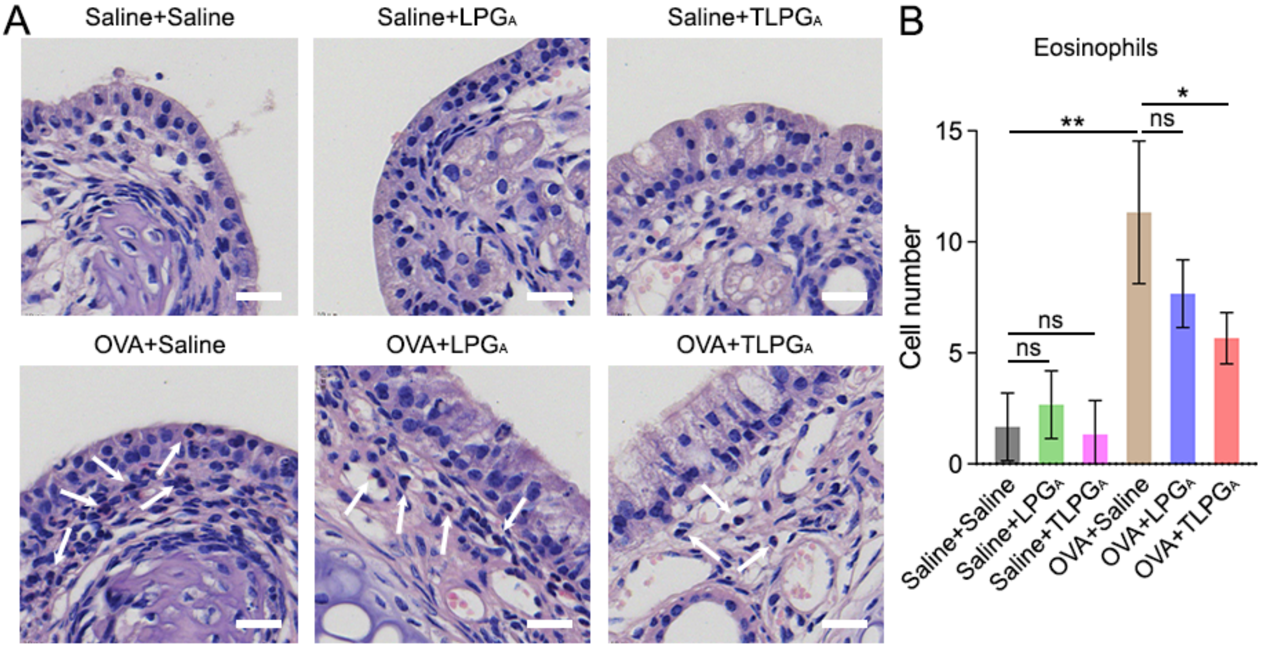

Figure S22. (A) Representative eosinophil staining images in the nasal mucosa of experimental mice. Scale bars: 20 μm. (B) Quantification of eosinophils in the staining images. Data represent mean ± S.D. (ns represents no significant difference, *p<0.05, **p<0.01).

19. Analysis of the inflammation in lungs

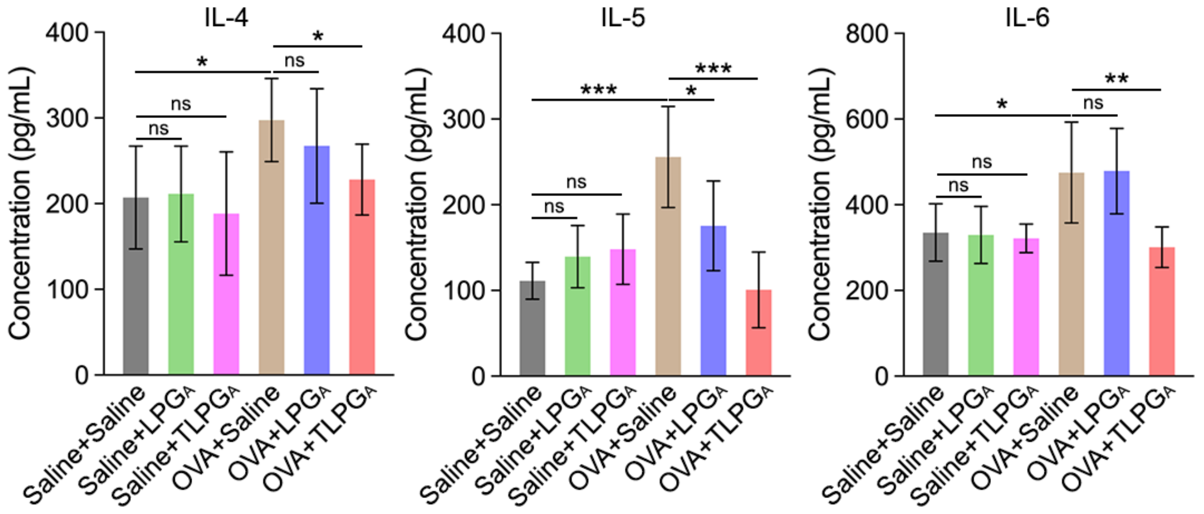

Figure S23. The IL-4, IL-5, and IL-6 concentration in BALF of mice following different treatments. Data represent mean ± S.D. (ns represents no significant, *p < 0.05, **p < 0.01, ***p < 0.001).

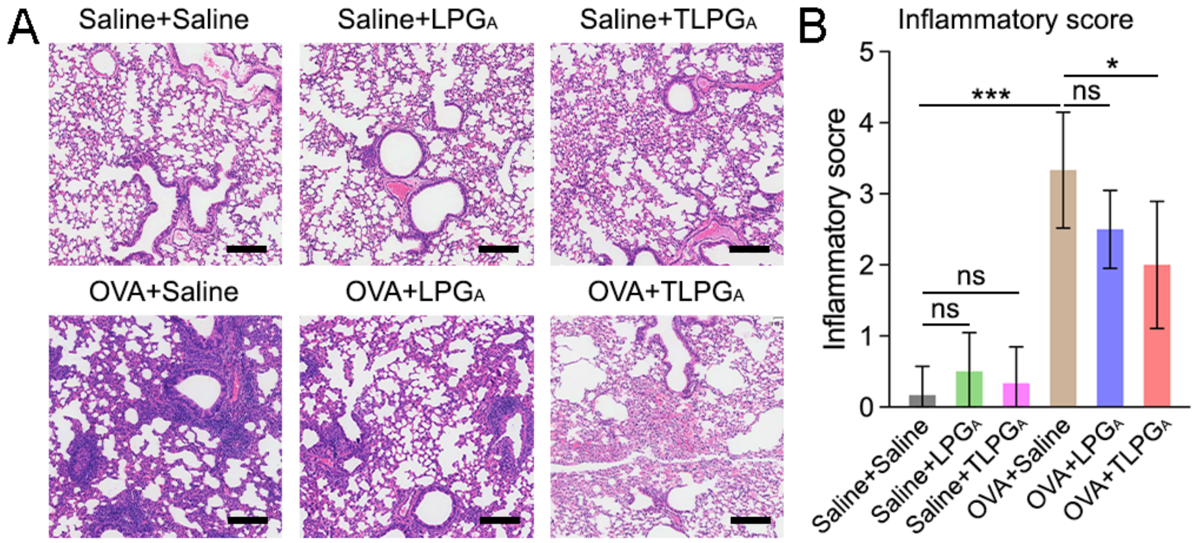

Figure S24. (A) Representative H&E staining images of lungs from experimental mice. Scale bars: 200 μm. (B) Quantification of inflammatory scores in the H&E staining images. Data represent mean ± S.D. (ns represents no significant difference, *p<0.05, **p<0.01, ***p<0.001).

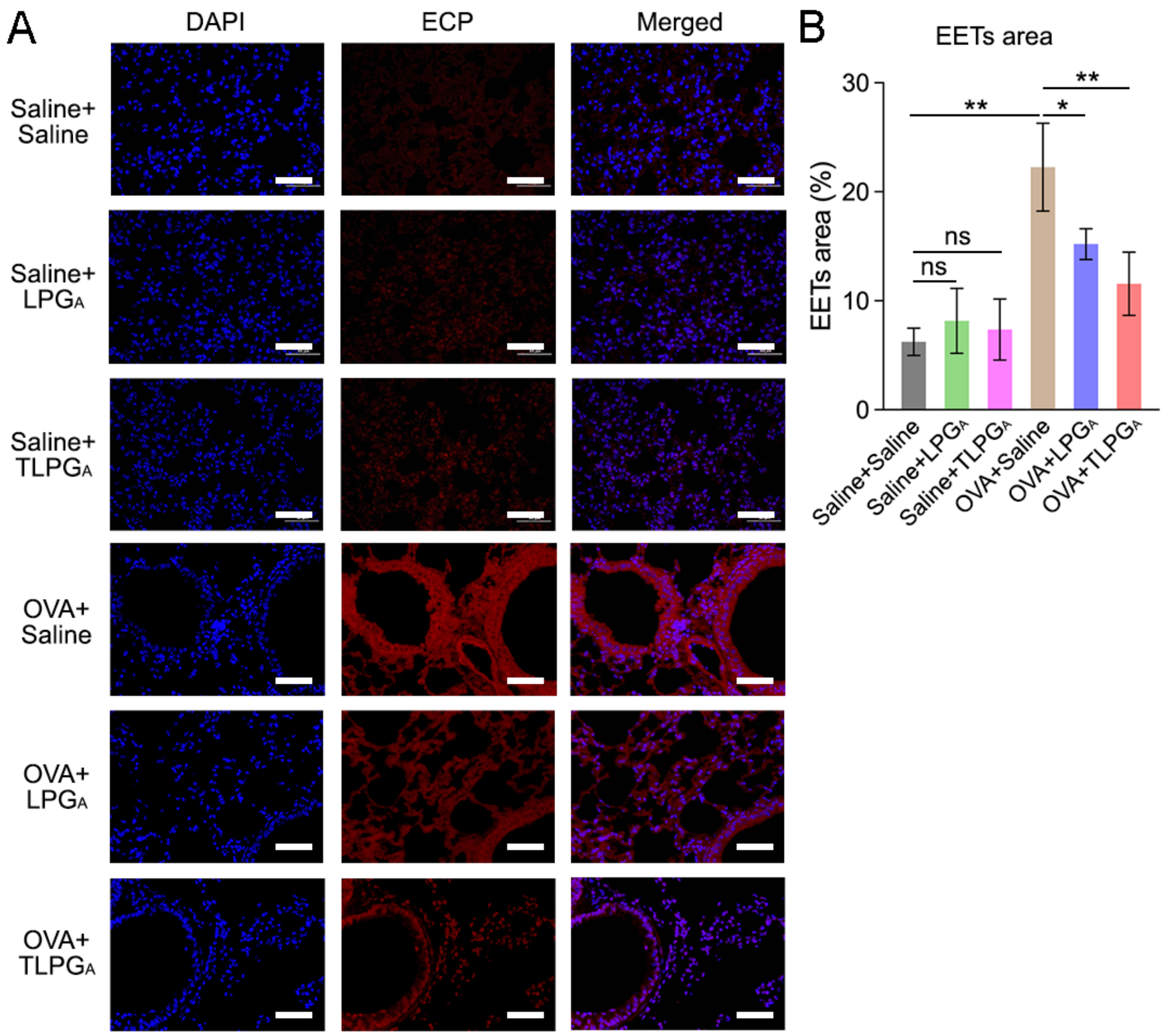

Figure S25. (A) Representative ECP staining images of lungs from experimental mice. Scale bars: 50 µm. (B) Quantification of EETs area in the ECP staining images. Data represent mean ± S.D. (ns represents no significant difference, *p<0.05, **p<0.01).

20. In vivo biocompatibility tests

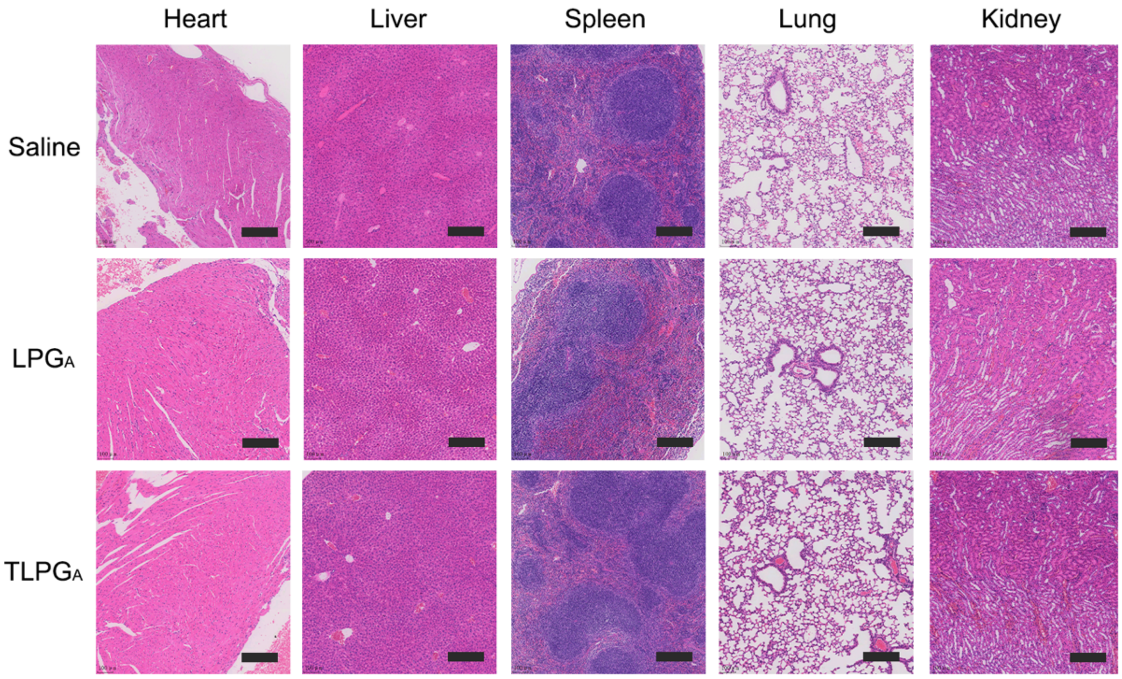

Figure S26. H&E staining of hearts, livers, spleens, lungs, and kidneys from experimental mice at 14th d after intranasal treatment with saline, LPG_A, and $TLPG_A$. Scale bars, 200 µm.

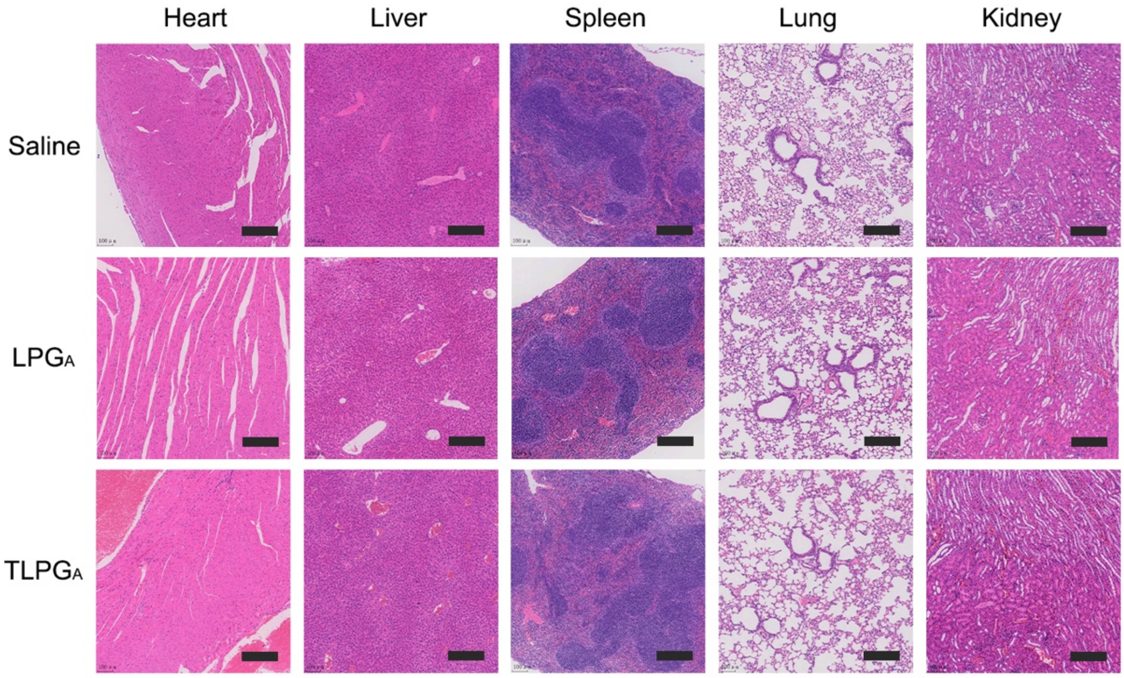

Figure S27. H&E staining of hearts, livers, spleens, lungs, and kidneys from experimental mice at 28th d after intranasal treatments with saline, LPG_A, and $TLPG_A$. Scale bars, 200 µm.

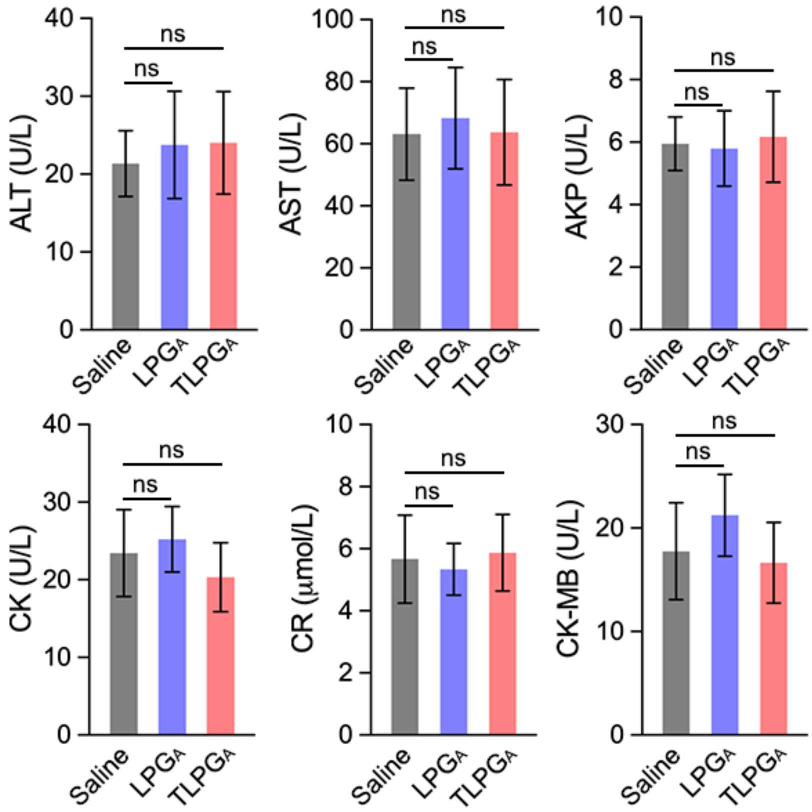

Figure S28. Biochemical parameters including ALT, AST, AKP, CK, CR, and CR-MB of serum from experimental mice at 28th d after intranasal treatments with saline, LPG_A, and $TLPG_A$. Data represent mean ± S.D. (ns represents no significant difference).

21. References

[1] F. Liu, S. Sheng, D. Shao, Y. Xiao, Y. Zhong, J. Zhou, C. H. Quek, Y. Wang, J. Dawulieti, C. Yang, H. Tian, X. Chen, K. W. Leong, *Matter* **2021**, *4* (11), 3677, https://doi.org/https://doi.org/10.1016/j.matt.2021.09.001.

[2] Y. Chen, Y. Wang, X. Jiang, J. Cai, Y. Chen, H. Huang, Y. Yang, L. Zheng, J. Zhao, M. Gao, *Bioactive Materials* **2022**, *18*, 409, https://doi.org/https://doi.org/10.1016/j.bioactmat.2022.03.028.

[3] K. Wu, X. Lu, Y. Li, Y. Wang, M. Liu, H. Li, H. Li, Q. Liu, D. Shao, W. Chen, Y. Zhou, Z. Tu, H. Mao, *Advanced science (Weinheim, Baden-Wurttemberg, Germany)* **2023**, *10* (23), e2300604, https://doi.org/10.1002/advs.202300604.

[4] J. Dawulieti, M. Sun, Y. Zhao, D. Shao, H. Yan, Y. H. Lao, H. Hu, L. Cui, X. Lv, F. Liu, C. W. Chi, Y. Zhang, M. Li, M. Zhang, H. Tian, X. Chen, K. W. Leong, L. Chen, *Science advances* **2020**, *6* (22), eaay7148, https://doi.org/10.1126/sciadv.aay7148.

[5] A. Gurtner, C. Borrelli, I. Gonzalez-Perez, K. Bach, I. E. Acar, N. G. Núñez, D. Crepaz, K. Handler, V. P. Vu, A. Lafzi, K. Stirm, D. Raju, J. Gschwend, K. Basler, C. Schneider, E. Slack, T. Valenta, B. Becher, P. Krebs, A. E. Moor, I. C. Arnold, *Nature* **2023**, *615* (7950), 151, https://doi.org/10.1038/s41586-022-05628-7.
